# Exploration of bacterial community classes in major human habitats

**DOI:** 10.1186/gb-2014-15-5-r66

**Published:** 2014-05-07

**Authors:** Yanjiao Zhou, Kathie A Mihindukulasuriya, Hongyu Gao, Patricio S La Rosa, Kristine M Wylie, John C Martin, Karthik Kota, William D Shannon, Makedonka Mitreva, Erica Sodergren, George M Weinstock

**Affiliations:** 1 The Genome Institute, Washington University, St Louis, MO 63108, USA; 2 Medical School of Indiana University at Indianapolis, Indianapolis, IN 46202, USA; 3 Washington University School of Medicine, Biostatistics in Medicine, St Louis, MO 63110, USA; 4 Department of Pediatrics, Washington University School of Medicine, St Louis, MO 63110, USA; 5 Current address: The Jackson Laboratory for Genomic Medicine, c/o University of Connecticut Health Center, 263 Farmington Avenue. Administrative Services Building, Call Box 901, Farmington, CT 06030, USA

## Abstract

**Background:**

Determining bacterial abundance variation is the first step in understanding bacterial similarity between individuals. Categorization of bacterial communities into groups or community classes is the subsequent step in describing microbial distribution based on abundance patterns. Here, we present an analysis of the groupings of bacterial communities in stool, nasal, skin, vaginal and oral habitats in a healthy cohort of 236 subjects from the Human Microbiome Project.

**Results:**

We identify distinct community group patterns in the anterior nares, four skin sites, and vagina at the genus level. We also confirm three enterotypes previously identified in stools. We identify two clusters with low silhouette values in most oral sites, in which bacterial communities are more homogeneous. Subjects sharing a community class in one habitat do not necessarily share a community class in another, except in the three vaginal sites and the symmetric habitats of the left and right retroauricular creases. Demographic factors, including gender, age, and ethnicity, significantly influence community composition in several habitats. Community classes in the vagina, retroauricular crease and stool are stable over approximately 200 days.

**Conclusion:**

The community composition, association of demographic factors with community classes, and demonstration of community stability deepen our understanding of the variability and dynamics of human microbiomes. This also has significant implications for experimental designs that seek microbial correlations with clinical phenotypes.

## Background

Knowledge of the composition, distribution and variation of bacteria in the human body has grown dramatically in the past decade. Different human habitats are composed of distinct microbial populations [1-8]. The range of abundance of components of the human microbiome extends over many orders of magnitude [9]. Inter-subject variation in bacterial community structure is also extensive in healthy humans [4,7]. Determination of the extent of the variability of the human microbiome is, therefore, crucial for understanding the microbiology, genetics, and ecology of the microbiome as well as for practical issues in experimental design and interpretation of clinical studies. In addition, the human microbiome is subjected to a continual flux of organisms from air, food, and other sources, transfer of organisms between body habitats through routine activity, cyclical changes in the physiology of body habitats on daily, monthly, and other timescales, which create changing selective pressures for each organism. Thus, temporal changes in bacterial communities (community stability) are also an important component of microbiome variation.

Evaluation of inter-subject variation is the first step to understanding the bacterial distribution in the human population. Furthermore, categorization of subjects based on the similarity or dissimilarity of their microbiota into groups by clustering techniques will not only help to reveal the bacterial distribution pattern in the population, but also facilitate our understanding of the underlying causes or the clinical association of specific types of microbial distributions. Indeed, recent data suggest the feasibility of such operational clustering. Specifically, the vaginal flora of asymptomatic women identified five groups by hierarchical clustering [6,10]. The five groups were defined based on the species or genera they contained. Race was found to be associated with the groups. Similar studies on microbial populations in stool samples identified three enterotypes [11]. Another approach identified two stool enterotypes by k-means clustering and found that long-term diet was associated with enterotypes [12], which emphasized the biological significance of these enterotypes. Old Amish stool microbiota is disproportionally of the *Prevotella* enterotype [13]. Enterotypes have been discovered not only in humans, as recent studies have described two and three enterotypes in mice and chimpanzees, respectively, which resembled the human stool enterotypes [14,15].

Several studies have not favored the enterotype concept [16-18]. Those studies focused on the stool microbiome distribution pattern in the human population, concluding that the stool microbiota was not a discrete distribution (three or two enterotypes) but rather a smooth gradient. Another issue is the appropriate number of clusters in the enteric bacterial community, for example, two or three stool enterotypes. These discussions largely emphasize the technical challenges in clustering data, and de-emphasize the value of categorization, namely, to codify and simplify relationships in a complex system and explore sensible biological groupings. A recent investigation on enterotypes across all the human body using the Human Microbiome Project (HMP) data showed that the enterotypes were affected not only by the data structure, but also by the methods applied in the clustering, such as clustering algorithms and distance measures [17]. These issues of clustering methodology are not surprising, since similar issues were previously seen in comparisons of clustering approaches for microarray data [19-21].

Because previous enterotype analysis of the HMP data is more technically orientated [17], biological inferences from the categorization are limited. Here, we used HMP 16S rRNA gene data from over 200 subjects, 18 body sites and two time points to interrogate the associated biology and explore the potential underlying mechanisms of the groups generated by two widely used clustering approaches. Because ‘enterotype’ originally referred to the microbiota type in stool, here we use an ecological term, ‘community class’, to refer to the clusters we identified in different habitats based on our cluster identification criterion, and use the generic term ‘cluster’ to refer to the groups of bacteria that do did not meet our criterion. We have identified three stool enterotypes and various community classes in the other 17 body habitats from the HMP 16S rRNA gene data and metagenomic shotgun data. We found association of demographic factors with different community classes. Also, for the first time we systematically assessed the stability of community classes and compared the subject composition in each community class from different habitats.

## Results

### Identification of community classes in human microbiota

Currently, there is no uniform statistical approach to determine the presence and optimal number of clusters or community classes from metagenomic samples [6,11]. Multiple approaches are recommended because the clustering approach is sensitive to the data sets [17]. We explored the community classes in each body site using both hierarchical clustering (complete linkage) and fuzzy k-means clustering with Bray-Curtis distances. The optimal number of clusters for a given habitat was determined by the silhouette method, a criterion used to choose the optimal number of clusters in previous enterotype studies [10-12]. The silhouette value is a measure of within and between cluster similarities. The number of clusters with the highest silhouette value is the optimal number of clusters in a data set. Twelve of eighteen sites had equal or higher silhouette values using hierarchical clustering with complete linkage, compared with k-means clustering (Table S1 in Additional file 1). The cluster similarities between different approaches and linkages used for hierarchical clustering are summarized in Table S2 in Additional file 1.

**Figure 1:**
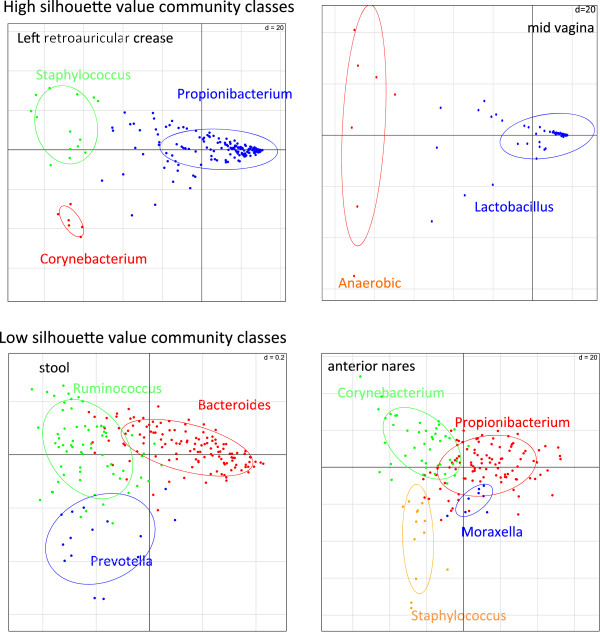
**Examples of community classes of human body habitats as illustrated by PCoA.** PCoA was used to visualize the community classes in different habitats. Samples are color-coded according to their community classes within the habitat. Only the major community classes from retroauricular crease are colored. The community classes show better separation in retroauricular crease and vaginal sites with higher silhouette values, and a less clear separation in stool and anterior nares with lower silhouette values.

Three community classes from stool were determined based on the averaged silhouette statistics (silhouette = 0.25 for three clusters). This clustering solution resulted in clusters of size N = 128, N = 15, and N = 66, which correspond to the *Bacteriodes*, *Prevotella* and *Ruminococcus* enterotypes identified from 39 European subjects [11], referred to as MetaHIT, as illustrated by principal coordinate analysis (PCoA) (Figure 1). It should be noted that the silhouette value only differs by 0.04 between two and three enterotypes.

To determine the effect of sample size on the number of clusters, we randomly subsampled 50, 100, 150 and 180 samples 100 times from the total 209 stool samples and computed the frequency of 2, 3, 4, and 5 clusters as the optimal cluster number in hierarchical clustering. At the subject scale, 52 of 100 subsamplings supported 2 clusters as optimal. With increasing numbers of subjects the frequency that 3 clusters was the optimal number increased to 72 of 100 subsamplings (Figure S1 in Additional file 2). This suggests that sample size as well as subject composition affects the number of clusters.

To further test the reliability of the clusters generated, we used two external confirmation methods. First, we clustered our data using an alternative approach. The 209 HMP stool samples were clustered using k-medoids with Jensen-Shannon divergence. This approach was identical to the approach used by the MetaHIT group [11], and generated the three clusters they identified with the highest silhouette values = 0.17 (Figure S2 in Additional file 2). The silhouette values for three clusters in the k-means clustering were less than those generated by hierarchical clustering (0.17 versus 0.25), suggesting that the latter technique performs better with the stool data type.

Although the same three enterotypes were generated by both clustering approaches, the prevalence of the specific enterotypes among the sampled subjects differed. Using hierarchical clustering, 61.2%, 31.6% and 7.2% of the stool samples were assigned to *Bacteroides*, *Ruminococcus* and *Prevotella* community classes, respectively. Using the MetaHIT approach, 47.4%, 42.1% and 10.5% of the stool samples were grouped to the above community classes.

To further confirm the validity of the clusters, whole genome shotgun (WGS) sequencing was conducted on 81 of the 209 samples. Applying our clustering/silhouette process to the metagenomic shotgun data recapitulated the same number and type of clusters as found with the larger sample size 16S rRNA gene and the MetaHIT data. Hence, the clusters were not due to bias from the amplification step of the 16S rRNA gene sequencing protocol.

Community classes were similarly determined for the rest of the 17 habitats. Silhouette values are similar for different numbers of clusters tested in the skin and vaginal sites (Table S1 in Additional file 1). For example, the average silhouette values for two to nine clusters in the right retroauricular crease ranged from 0.51 to 0.52. Although the high silhouette values suggest that there were true clusters in this habitat, the optimal number of cluster was undetermined. The structure of this habitat always involved a single large cluster with high silhouette value and a variable number of smaller clusters (Figure S3 in Additional file 2), and the indeterminacy was due to estimating the number of these smaller clusters. Manual inspection of clusters determined the optimal number of clusters under this condition: placement of 158 samples from the right retroauricular crease in one cluster produced a very high silhouette value (0.59), while placement of 31 samples in the other cluster resulted in a silhouette value of 0.15. Further dividing of the 31 samples into two clusters resulted in much higher silhouette values (0.49) for the 19 samples in the first cluster, but an even lower value for the 12 remaining subjects (0.007). The latter value suggests that the bacterial community structures from the group of 12 subjects were very heterogeneous, prompting assignment of these samples as different clusters. Based on the above inspection of the silhouette values from individual groups and the taxonomic profiling from the dendrogram (Figure S6 in Additional file 2), we chose six clusters as the optimal number for the right retroauricular crease. Most subjects had high silhouette values for the six clusters, suggesting the cluster solutions are appropriate. A subset of samples had negative silhouette values, indicating improper grouping. For example, the subject with a negative silhouette value at the top of Figure S3 in Additional file 1 was grouped into *Staphylococcus* community classes with 16% of the *Staphylococcus* and 68% of *Helicobacter*. The relative abundances of *Helicobacter* in the rest of the subjects are low (<1%); thus, this subject is inappropriate to be grouped in any clusters. In general, subjects with negative silhouette values are regarded as outliers. These subjects with unique bacterial community structure are not surprising considering the heterogeneity of the skin microbiota. Manual inspection, following the logic described above, was necessary for the three vaginal sites and four skin sites where silhouette values were similar between two or more clusters.

Table 1 summarizes the optimal number of community classes, corresponding silhouette values, and the number of subjects in each community class. Silhouette values for the optimal number of clusters vary by habitat. Three to six classes for skin sites and two to three classes for the vagina were identified with high silhouette values (>0.5), indicating that community classes at these sites were well-defined (Figure 1) [22]. We also identified the five community classes (four driven by the genus *Lactobacillus* and one by anaerobic genera) in a subset of posterior fornix samples using WGS data, as previously reported [6,10]. Strain level analysis achieved finer resolution. The *Lactobacillus gasseri* group was divided into two subgroups occupied by the same species but different strains of *L. gasseri* (Figure S4 in Additional file 2). Interestingly, two anaerobic community classes were identified in the posterior fornix: *Gardnerella* dominated one community class, while *Prevotella* and *Atopobium* dominated the other. Although it was well known that all three genera were associated with vaginosis, our result calls attention to further categorization of these bacteria in healthy subjects as well.

**Table 1:** Summary of community classes and their stability in human habitats

**Habitat type**	**Habitats**	**Silhouette value**	**Clusters**	**Membership**	**Dominant genera**	**ARI for two visits**
**Type I**	Posterior fornix	0.86	3	3/5/80	*Gardnerella / Prevotella / Lactobacillus*	0.57
	Mid-vagina	0.78	2	7/81	Anaerobic genera */ Lactobacillus*	0.58
	Vaginal introitus	0.66	2	13/67	Anaerobic genera */ Lactobacillus*	0.44
	Left retroauricular crease	0.6	3	6/14/158	*Corynebacterium / Staphylococcus / Propionibacterium*	0.56
	Right retroauricular crease	0.52	6	4/4/4/4/19/154	*Neisseriaceae_Unclassified*^a^*/ Corynebacterium / Pelomonas / Anaerococcus / Staphylococcus / Propionibacterium*	0.47
**Type II**	Left antecubital fossa	0.30	5	2/6/7/28/30	*Sporacetigenium / Staphylococcus / Ralstonia/ Propionibacterium / Corynebacterium*	0.07
	Right antecubital fossa	0.37	6	3/3/4/6/8/61	*Streptophyta / Corynebacterium / Staphylococcus / Streptococcus / Haemophilus*^b^*/ Propionibacterium*	0.16
	Keratinized gingiva	0.32	2	59/140	*Prevotellaceae_Unclassified / Streptococcus*	0.37
	Buccal mucosa	0.28	2	32/153	*Haemophilus / Streptococcus*	0.12
	Hard palate	0.25	2	10/183	*Veillonella /Streptococcus*	0.04
	Anterior nares	0.24	4	8/13/44/101	*Moraxella / Staphylococcus / Propionibacterium / Corynebacterium*	0.26
	Stool	0.25	3	15/66/128	*Prevotella / Ruminococcaceae*^a^*/ Bacteroides*	0.26
	Tongue dorsum	0.18	3	35/77/94	NA	
	Subgingival plaque	0.21	2	97/104	NA	
	Throat	0.19	2	91/93	NA	
	Supragingival plaque	0.15	2	97/111	NA	
	Palatine tonsils	0.17	2	96/103	NA	
	Saliva	0.14	2	89/93	NA	

^a^*Neisseriaceae* in retroauricular crease and *Ruminococcaceae* in stool are the dominant orders (not genera).

^b^A heterogeneous group.

ARI, Adjusted Rand Index; NA, not applicable (indicator genera are not shown because of very low silhouette value).

The remaining habitats presented relatively low silhouette values. Four community classes were identified from 166 anterior nares samples (silhouette = 0.24; Figure 1). Except for keratinized gingiva, buccal mucosa and hard palate, the silhouette values in the rest of the oral sites were <0.2, suggesting that the bacterial communities in these habitats were more homogenous [23]. However, biologically interesting community classes were identifiable in some of the habitats with silhouette values <0.2, as addressed below.

The number of community classes was not consistent among similar sites. Symmetric sites, the left and right retroauricular creases, have three and six community classes, respectively, while the left and right antecubital fossas have five and six community classes, respectively. However, both retroauricular crease sites contained the same dominant community classes defined by *Corynebacterium*, *Staphylococcus* and *Propionibacterium*. The difference in the less dominant community classes is partly because the source subjects for the left and right site samples did not completely overlap. The high degree of inter-variation among the skin microbial community can produce some unique community classes in a small subset of subjects. The three vaginal sites are proximal, but contained different numbers of community classes. Most subjects were dominated by *Lactobacillus*. A small proportion of the subjects were dominated by one or two groups of anaerobic genera.

### Habitat classification and indicator taxa of community classes

In the high silhouette value habitats (>0.5), such as the retroauricular crease, many samples from the main community class (*Propionibacterium* community class) were tightly clustered, while the remainder of the samples formed smaller community classes, in which only a few subjects were included (Figure 1). On the other hand, in community classes in the relatively low silhouette value habitats (0.25 to 0.5), such as stool and anterior nares, samples were less tightly clustered than for the high silhouette value habitats.

To examine the structure underlying different community classes, we correlated the alpha diversities (number of taxa within a sample) and the silhouette values in the 18 habitats. As indicated by Figure S5 in Additional file 2, silhouette values are strongly negatively correlated with the Shannon diversities (Pearson correlation = 0.96). In particular, the two lowest alpha diversity habitats, vagina and retroauricular crease, had the highest silhouette values of the clusters, and the high alpha diversity habitats (saliva) exhibited low average silhouette values of its clusters. Based on the alpha diversity and silhouette values, the 18 habitats were divided into two types: type I (low diversity with median Shannon index <1.5 and high silhouette value) and type II (high diversity and low silhouette values) habitats. Type I habitats with low alpha diversity (Figure 1) were dominated by one genus. For example, *Lactobacillus* was present in each of the vaginal samples with an average abundance of 92% in the tightly clustered subgroup. Alternatively, bacterial communities in type II habitats were dominated by different genera to different degrees, leading to highly diverse communities. This diversity was reflected in the clusters, where samples were less tightly clustered than in type I habitats (Figure 1).

The definition of community class is based on the relative abundance of genera in bacterial communities. Certain key taxa are assigned as indicators, whose presence, absence, and relative abundance characterize that community class [24]. Indicator taxa were determined using the Dufrene-Legendre Indicator Species approach [25].

#### Type I habitats

**Figure 2:**
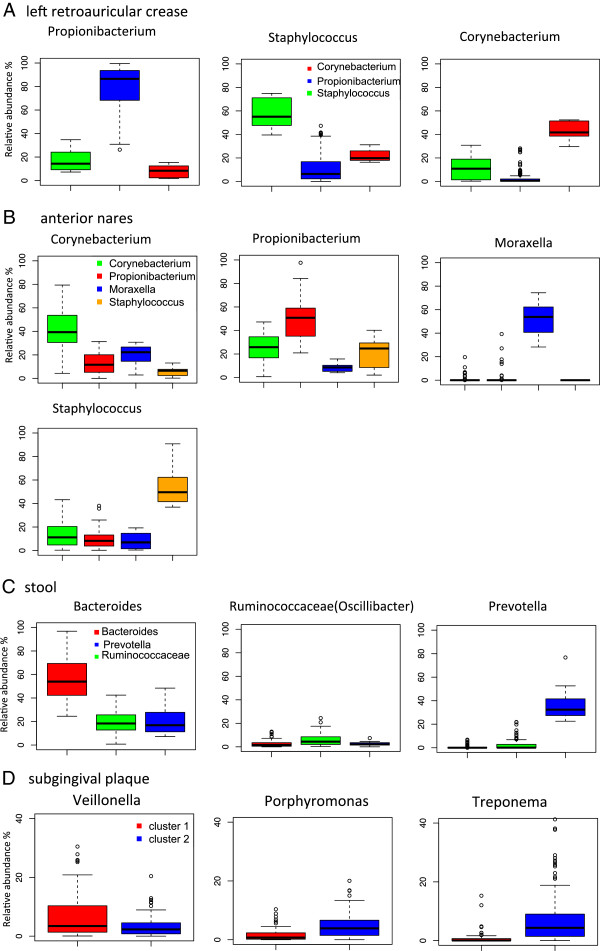
**Examples of indicator taxa between community classes.** Indicator taxa driving the differentiation of community classes were identified using the indval function in the labdsv package in R. Boxplots are labeled according to the dominant taxa in the community class. **(A-D)** The colors represent different community classes. The relative abundances of dominant taxa (y-axis) in each community class are plotted for the type I habitat left retroauricular crease **(A)**, and the type II habitat anterior nares **(B)**, stool **(C)**, and subgingival plaque **(D)**. In **(D)**, the name of the community classes are designated as a generic term, cluster 1 or cluster 2, because of the very low silhouette value (<0.2).

The left and right retroauricular crease share three of the same three community classes (*Propionibacterium*, *Staphylococcus*, *Corynebacterium*) and most subjects were found in these three community classes. We used the left retroauricular crease as an example to show the indicator taxa that differentiate the community classes. While *Propionibacterium* is ubiquitously present on the skin of the healthy population, the median relative abundance is 86.5% in the *Propionibacterium* community class, but only 8.3% and 11.4% in the other community classes (Figure 2A). Likewise, the *Staphylococcus* community class features high abundance *Staphylococcus* (median relative abundance 55.0%), compared with 6.4% and 19.8% in the other community classes. The median relative abundance of *Corynebacterium* is 41.7% in the *Corynebacterium* community classes and 0.6% and 10.8% in other classes. Differentiation of community classes also involves less abundant taxa. In total, 14 taxa were significantly different between the community classes of the left retroauricular crease samples (*P* < 0.01; Table S3 in Additional file 1).

Using mid-vagina as a vaginal habitat representative, we determined indicator genera for the vaginal community classes. The relative abundance of *Lactobacillus* ranges from 31.3% to 99.9% in the *Lactobacillus* community class. In the anaerobic community class, *Lactobacillus* is less abundant while the anaerobic genera, such as *Prevotella*, *Sneathia*, *Bifidobacterium*, *Megasphaera*, *Dialister*, and *Atopobium*, are more abundant (Table S3 in Additional file 1). These genera are reported to be vaginosis-associated genera [26].

#### Type II habitats

The top four most abundant genera in the anterior nares are *Corynebacterium*, *Propionibacterium*, *Staphylococcus*, and *Moraxella*, each of which is the dominant genus for a distinct community class (Figure 2B; Table S3 in Additional file 1). Each of these genera contains pathogenic species, suggesting that the anterior nares is a potential reservoir for pathogens.

Characterization of anterior nares bacterial communities in 40 individuals by 16S rRNA fingerprinting based on single-strand conformation polymorphisms demonstrated 10 clusters with the majority of subjects (36 of 40) grouped into five clusters [27]. Four of these clusters were identified in our analysis, the exception being a *Finegoldia* group. In our 16S rRNA gene data set *Finegoldia* was present in only one of the samples, and with very low abundance. *Finegoldia* is isolated most frequently from various infected sites [28], and is less common in healthy subjects.

Five and six community classes were identified in the left and right antecubital fossa samples, respectively. Three of these, dominated by *Propionibacterium*, *Corynebacterium*, and *Staphylococcus*, were also found in the retroauricular crease samples. However, *Propionibacterium* abundance in the *Propionibacterium* community class of antecubital fossa is lower than that in retroauricular crease (average of 61.6% of the community compared with 92%), it is still 5-fold more abundant than in the other community classes. In total, 24 indicator genera were identified among the 7 community classes (Table S3 in Additional file 1).

In the MetaHIT study, the *Ruminococcus* type is the most frequent enterotype in stool, followed by *Bacteroides* and *Prevotella*[11]. In our analysis, most of the subjects were grouped to *Bacteroides* followed by *Ruminococcus* and *Prevotella*. The average abundance of *Bacteroides* in our data set is 55%, in contrast to the average abundance of 35% in the other study (Figure 2C) [11]. There are methodological as well as demographic differences in the subjects in these studies, so the quantitative differences are not surprising.

It is difficult to delimit clusters in most oral sites due to the homogeneity of bacterial communities. We nevertheless observed biologically important groups in the subgingival plaque in this healthy cohort. We found that the subgingival plaque from 122 subjects was inhabited by periodontitis-associated genera such as *Treponema* and/or *Porphyromonas* with a median relative abundance of 4.3% and 3.9%, compared with 0.05% and 0.73% in the rest of the subjects, respectively. Fourteen subjects exhibited significant amounts of *Treponema* and/or *Porphyromonas*, accounting for 26% to 44% of total bacteria in their subgingival plaque. Those subjects are major components of cluster 2 in Figure 2D. We use the generic term cluster in this context to distinguish the community classes described in other habitats where clusters have a higher silhouette value. On the other hand, the relative abundance of *Veillonella*, a genera that slows the development of dental caries [29], was very low in cluster 2 (Figure 2D). In the supragingival plaque, pathogenic anaerobes are less abundant compared with subgingival plaque because this is a less anaerobic environment compared with the subgingival region [30].

In keratinized gingivae, buccal mucosa, hard palate, palatine tonsils and throat, one community class was dominated by *Streptococcus*, and the other community class was dominated by genera varying with habitats (Table S3 in Additional file 1). Interestingly, 59 subjects had keratinized gingiva microbiome distinguished by a high abundance of unclassified *Prevotellaceae* and unclassified *Bacteroidales*; this community class represented a less characterized taxonomic group (Figure S6 in Additional file 2). Tongue dorsum and saliva are two habitats in which the *Streptococcus* abundance is less dominant than in other oral sites. Instead, *Neisseria* is an essential genus in one-third of tongue dorsum samples, resulting in detection of the *Neisseria* community class (Table S3 in Additional file 1; Figure S6 in Additional file 2).

### Community class comparisons across all habitats

The HMP data sets not only provide the opportunity to characterize the community classes of multiple habitats, but also allow us to determine if there are any correlations between community classes at different habitats in the same individual. This can be addressed by determining if a group of subjects who carry a particular community class in one habitat also belong to the same community class at other habitats.

**Figure 3:**
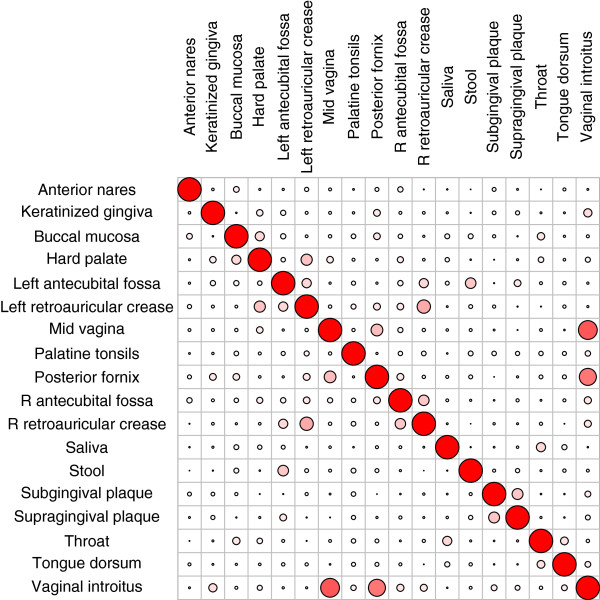
**Community class comparisons across all habitats.** Cluster similarity was analyzed using the ARI. A large overlap of carriers was detected between community classes in left and right retroauricular creases as well as within three vaginal sites. The color saturation and size of the circles represent the degree of cluster similarity. The circles along the diagonal from top left to bottom right, with an ARI equal to 1, indicate self-comparison.

To answer this question, we compared the subjects from different community classes in each pair of habitats. Subjects who had samples from both habitats were included in the clustering analysis. The Adjusted Rand Index (ARI) was used to assess the similarity between clusters in different habitats [31], where a comparison is made between the assignments of each pair of subjects in the clusters of the two habitats under comparison. Complete correlation between subjects at two habitats produces an ARI of 1. As shown in Figure 3, the subject composition in the community classes was highly consistent, with ARI = 0.64 in the three vaginal sites. The left and right retroauricular creases were also similar when comparing subject compositions in the community classes (ARI = 0.32). Other paired habitats showed low similarity for community classes (Figure 3).

Because the anterior nares, retroauricular crease, and antecubital fossa each have three community classes dominated by *Propionibacterium*, *Corynebacterium*, and *Staphylococcus*, we performed a detailed comparison of subjects carrying these three community classes. In particular, the *Corynebacterium* community class is the most common community class in the anterior nares, yet only 18% (16 of 87) of samples with the *Corynebacterium* community class in the anterior nares belonged to the *Corynebacterium* community class of antecubital fossa. In contrast, 50% of the samples with the *Corynebacterium* community class in the anterior nares were assigned to the *Propionibacterium* community class of antecubital fossa. The rest were assigned to the *Staphylocccus* community class or other small classes in antecubital fossa. *Moraxella* was the fourth community class in anterior nares, and subjects with a high abundance of *Moraxella* in their anterior nares did not have high abundance *Moraxella* in their skin. Therefore, bacterial community structures across subjects are confined to a given habitat and the drivers of community structure act independently of other unrelated habitats.

### Associations of demographic factors with bacterial community structure

The association of demographic factors (gender, geographical location, ethnicity, body mass index (BMI), age) with each community class was tested by Fisher’s exact test or ANOVA, and *P*-values were corrected using the Bonferroni approach (Materials and methods; Table 2; Figure S6 in Additional file 2).

**Table 2:** Association of demographic factors with community classes

	**Gender**		**Age**		**Race**		**Sites**		**Vaginal PH**	
**Habitats**	**Community classes for comparison**^**a**^	***P***^**b**^	**Community classes for comparison**^**a**^	***P***^**b**^	**Community classes for comparison**^**a**^	***P***^**b**^	**Community classes for comparison**^**a**^	***P***^**b**^	**Community classes for comparison**^**a**^	***P***^**b**^
**Anterior nares**	*Propionibacterium Corynebacterium*, *Staphylococcus*, *Moraxella*	0.05								
**Antecubital fossa**	*Staphylococcus*, *Propionibacerium*	0.005								
**Retroauricular crease**	*Staphylococcus*, *Propionibacterium*, *Corynebacterium*	0.0004	*Propionibacterium* non-*Propionibacterium*	0.003						
**Hard palate**			*Veillonella*, *Streptococcus*	0.02						
**Buccal mucosa**							Haemophilus Streptococcus	0.00005		
**Subgingval plaque**							Two clusters	8.0 × 10^-10^		
**Supragingival plaque**					Two clusters	0.03	Two clusters	3.8 × 10^-7^		
**Throat**					Two clusters	0.04	Two clusters	6.2 × 10^-6^		
**Palatine tonsils**					Two clusters	0.03	Two clusters	9.5 × 10^-7^		
**Saliva**					Two clusters	0.03	Two clusters	0.00005		
**Vagina**					*Lactobacillus*, anaerobic class	0.04			*Lactobacillus*, anaerobic class	0.01

^**a**^Community classes used in the demographic association analysis.

^**b**^*P*-values after Bonferroni correction.

Empty fields indicate there is no significant difference between the community classes for a give demographic factor.

**Figure 4:**
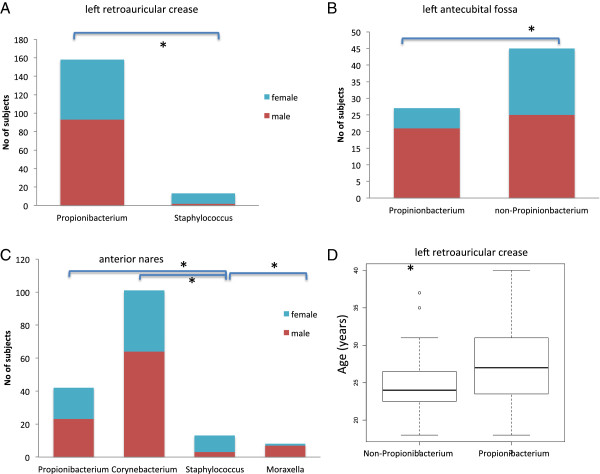
**Examples of demographic factor correlations with community classes. (A)** Gender differences in retroauricular crease samples, with females more prevalent in the *Staphylococcus* community class. **(B)** Gender differences in community classes of antecubital fossa. The *Propionibacterium* community class has significantly more males than females while the non-*Propionibacterium* community classes have significantly more females than males. **(C)** The *Staphylococcus* community class in anterior nares is dominated by females, and the female proportion is significantly higher than in the other three community classes. **(D)** The ages of subjects in the *Propionibacterium* community class and the other community classes of retroauricular crease are significantly different. Asterisks indicate statistically significant differences.

Gender was significantly different between the community classes in the retroauricular crease, antecubital fossa and anterior nares (Figure 4). In the retroauricular crease, the *Staphylococcus* community class was mainly carried by females in contrast to the relatively even gender distribution for *Propionibacterium* and *Corynebacterium* community classes (*P* = 0.0004; Figure 4A). In the antecubital fossa, male samples were dominated by *Propionibacterium* and females by the *Staphylococcus* community class (*P* = 0.005; Figure 4B). In anterior nares, female subjects had over-representation of *Staphylococcus* community classes in anterior nares, whereas the *Moraxella* community class was mainly composed of male subjects (*P* = 0.05; Figure 4C). As expected, the relative abundances of each genus described above in male and female subjects from skin and anterior nares sites were also significantly different (Figure S7 in Additional file 2).

Among the 15 subjects with the *Prevotella* enterotype in stool, 13 were male, which did not differ from the gender distributions of *Bacteroides* and *Ruminococcus* enterotypes after Bonferroni correction (*P* = 0.14). However, among samples in which *Prevotella* was present, we found that the median abundance of *Prevotella* in all the male samples was 10-fold greater than in female samples (*P* = 0.005; Figure S7 in Additional file 2). We did not detect a correlation of BMI with any enterotypes or with the ratio of *Bacteroidetes* and *Firmicutes* in stool.

Consistent with prior studies [6], pH was strongly associated with the bacterial community structure in the vaginal sites (Table 2). pH was significantly higher in the anaerobic bacteria-dominant community class (*P* = 0.01). Ethnicity differs significantly (*P* = 0.04) in the *Lactobacillus* community class compared to the anaerobic bacteria subgroup.

The subjects in the *Propionibacterium* community class are about 4 years older than other community classes in retroauricular crease (*P* = 0.003; Figure 4D). Age was also significantly different between community classes in hard palate (*P* = 0.02), as were site of residence (St Louis versus Houston) and race in oral sites (Table 2).

### Community class stability

**Figure 5:**
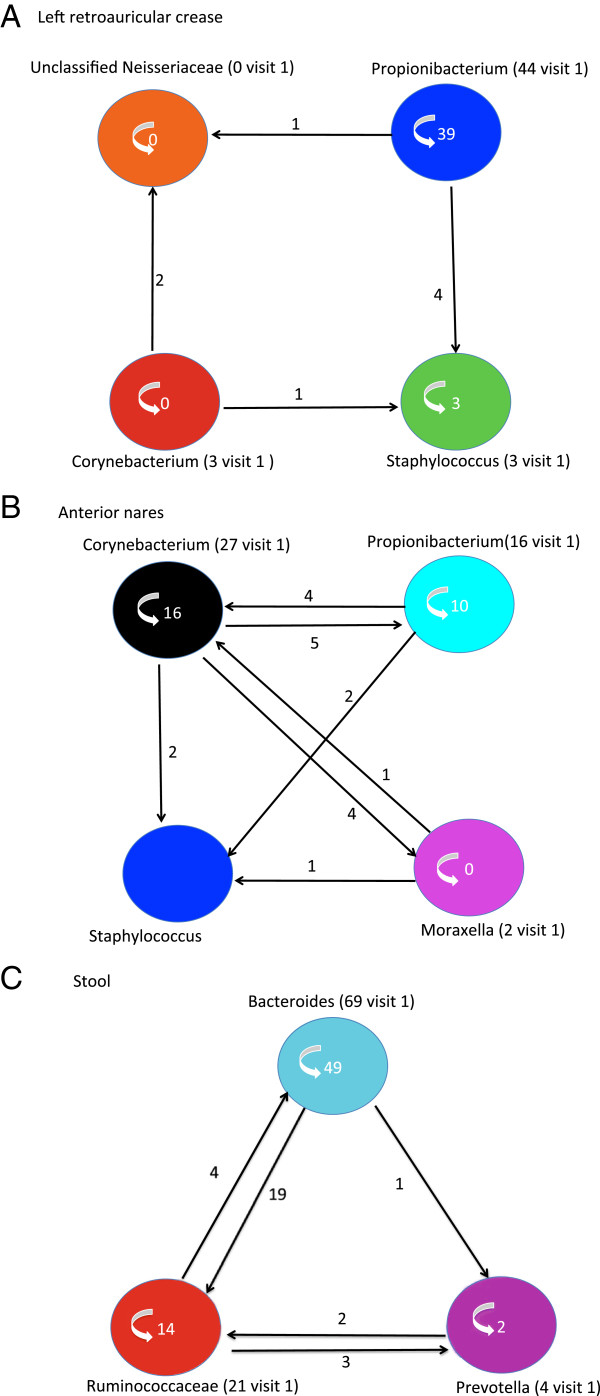
**Dynamics of community classes over time. (A-C)** Subjects from different community classes switch to other community classes or maintain their original community class in different habitats: **(A)** retroauricular crease, **(B)** anterior nares, and **(C)** stool. The colored circles represent different community classes. The names of community classes are followed by the number of subjects belonging to the community classes at the first visit. Subjects transferring to other community classes at the second visit are indicated by arrows along with the number of subjects that switched. The number inside each community class indicates the number of subjects maintaining the same community class at the second visit.

Figure 5A illustrates the community class changes between two time points using left retroauricular crease as an example. Three community classes were identified in left retroauricular crease in the first sampling and maintained in the second sampling. The ARI measuring the agreement of paired subjects in the clusters of two time points was 0.47. In particular, 86% of the subjects were in the same community class on both visits. This relative stability is largely because of the stability of the major *Propionibacterium* community class. Of 44 subjects, 39 maintained the *Propionibacterium* community class (Figure 5A). One of three subjects from the *Corynebacterium* community class switched to the *Staphylococcus* class while the three subjects in the *Staphylococcus* class still clustered in the same class. One subject from the *Propionibacterium* and two subjects from the *Corynebacterium* class switched to an unclassified *Neisseriaceae* class at the second time point. Because each community class was defined by the relative abundance of the genera in the community, switching between community classes indicates a significant change in the abundance of genera.

We also evaluated community class stability in the vaginal introitus. The ARI was high (0.57) for community classes from the two samplings. Eighty-six percent of subjects retained the same community classes between visits. Most subjects (23 of 25 subjects) from the *Lactobacillus* community class stayed in the same community classes at the second visit. Two subjects switched to the anaerobic community class. Two of four subjects from the anaerobic community class in the first visit switched to the *Lactobacillu*s class on the second visit (Figure S8 in Additional file 2). Subjects that switched from the *Lactobacillus* to the anaerobic community class showed increased vaginal pH, which may be caused by the decrease in lactic acid bacteria. Overall, the community classes in type I habitats (retroauricular crease and vagina) tend to be stable over time because of the high abundance of single genera in their communities.

In contrast to the type I habitats, community classes in type II habitats appeared to be less stable over time. For this analysis, the left and right antecubital fossa data were combined at each time point because of the small amount of repeat data available. The ARI was 0.2 for the antecubital fossa. Of 33 subjects, 20 (60%) maintained their community classes at the second visit (Figure S8 in Additional file 2). The less dominant community under active switching represented the less stable community class. Likewise, 22 of 48 sample pairs (45%) switched their anterior nares class at the second visit (ARI = 0.26), indicating the dynamic nature of the bacterial community in the anterior nares (Figure 5B). Thirty-two percent of subjects switched from *Corynebacterium* to *Propionibacterium* and 24% of *Propionibacterium* subjects switched to *Corynebacterium*. Subjects also switched in both directions between the *Corynebacterium* and *Moraxella* classes. Switching also was observed from *Propionibacterium* to *Staphylococcus*, *Staphylococcus* to *Corynebacterium*, and *Moraxella* to *Staphylococcus*. We did not observe switching from the *Staphylococcus* to the *Propionibacterium* or *Moraxella* classes. This may be because of the small sample size (two subjects) for the *Staphylococcus* community class. Also, it will be interesting to probe the role of gender barriers in the transition between community classes because male and female subjects were dominated by different community classes.

Switches between stool community classes (31%) were more frequent than in type I habitats, but less frequent than in other type II habitats (ARI 0.26). The switching mainly occurred between the *Bacteroides* and *Ruminococcus* community classes (Figure 5C). Although *Bacteroides* dominates in the *Bacteroides* community class, a subset of subjects had high relative abundance of *Ruminococcus*. These subjects tended to convert to the *Ruminococcus* community class. This is also true for subjects belonging to the *Ruminococcus* community class but with a relatively high abundance of *Bacteroides.* These subjects tended to convert to the *Bacteroides* community class at the second visit.

As addressed above, community classes in most oral habitats are not as distinct as type I habitats, the active conversion between classes reflecting the uncertainty of the clusters in those habitats except saliva, tongue dorsum and keratinized gingiva (Table 1).

We additionally examined the variation of *Treponema* between visits in subgingival plaque. The 14 subjects with high relative abundances of *Treponema* (26% to 44%) at the first visit retained 7- to 8-fold higher than average abundance of *Treponema* at the second visit. The stability of the high abundance *Treponema* phenotype between visits in a subset of the subjects shows that the predominance of *Treponema* in these individuals is not a transient event, reinforcing the concept that these subjects are at higher risk to develop periodontal disease.

## Discussion

Reports on the three stool enterotypes sparked an ongoing debate on whether stool microbiota is actually discrete or a continuum [16]. Enterotypes have been reported in humans and other animals [12,14,15]. There has been controversy regarding the conceptual appropriateness of discrete or categorical enterotypes versus a population description based on a gradient pattern of bacterial community structure [18]. The debate has been focused largely on the clustering techniques [17] and the assignment of the number of clusters in the community. However, these concerns overlook the purpose of the categorization of the microbiota in the human body, that is, determination of like versus not-like groupings of organisms, and easily conveyed descriptions of populations with which to find biologically meaningful groups. Clustering simplifies the complex relationships between objects and the cluster solutions vary with different distance/dissimilarity measurements and clustering algorithms [17,20,21]. Clustering is only an exploratory technique and should not play a decisive role in data analysis.

Our analysis of stool identified three stool enterotypes, consistent with previous studies. A study using the HMP metagenomic data indicated only two clusters for each of the 18 body sites, including stool [17]. Our analysis identified two clusters for oral and two vaginal sites, but more in stool and skin habitats. We further manually inspected the hierarchical cluster solutions in skin and vaginal sites where the silhouette values were similar for different numbers of clusters. This manual inspection resolved more biologically interesting clusters. This manual inspection is based on subjective interpretation and related biological knowledge is thus required. For example, our inference of two community classes in the posterior fornix was based on our findings from an independent project where race differed in different anaerobic groups (data not shown). Overall, the clustering analysis serves as a starting point for assessing the existence and number of the groupings, and obligate further investigation to determine biological validity.

Recent work has shown the effect of diet on enterotypes [12]. Specific long-term diets, especially those high in protein and fat, were linked to *Bacteroides* enterotypes, while carbohydrates were linked to *Prevotella* enterotypes [12,15]. The predominance of the *Bacteroides* enterotype identified in the HMP healthy cohort may reflect the natural presence of enterotypes in St Louis and Houston populations on American diets. Our findings might have clinical relevance. For example, metabolic diseases such as obesity are associated with the elevation of a wide range of cytokine and inflammation markers. Calprotectin, a gut inflammation marker, is higher in mice that harbor a *Bacteroides* enterotype [12,15], suggesting that this genus triggers low-grade inflammation reactions in the gut. The relationships of enterotypes, diet and inflammation raise the possibility of manipulating gut enterotypes to mitigate risk of metabolic diseases.

Subjects from the HMP cohort are healthy as defined by clinical criteria; we nevertheless detected signals for disease. For example, a cluster dominated by periodontitis-associated genera such as *Treponema* and/or *Porphyromonas* was identified in subgingival plaque samples. *Treponema* is considered a major etiological bacteria in periodontitis and *Porphyromonas* is strongly associated with chronic adult periodontitis [30,31]. *Treponema* is a genus consisting of many species, but the *Treponema* species are rare in healthy subjects compared with subjects with periodontal disease [32]. The high abundance of periodontal disease-associated genera and low abundance of protective genera in the healthy population is an example of the pathogenic bacterial load carried by healthy individuals. Similarly, the anaerobic group was identified in a small subset of the vaginal samples from this cohort. A high abundance of anaerobic genera in vagina is often linked to vaginosis. The identification of an anaerobic group in the HMP cohort may be a result of the inclusion of unhealthy individuals in the cohort, because of the incomplete criterion used for the diagnosis of bacterial vaginosis [32]. Alternatively, it may represent a normal vaginal flora for non-Caucasians [6].

Phenotypic characterization of community classes also sheds light on mechanisms underlying these differences. Gender correlated with community classes in the skin, anterior nares and stool. What drives the difference between male and female skin and nasal community composition? Intrinsic properties of bacteria and physiological differences between genders may contribute to the microbiota composition difference. Male skin has more collagen and sebum with larger pores, a richer blood supply, and an increased tendency to sweat [23,33]. Thus, male skin may provide more nutrition for the two slow-growing genera, *Propinionbacterium* and *Corynebacterium*, that are favored among men in this cohort. The different bacterial compositions of male and female skin might also lead to phenotypic differences. For example, odor precursors in men and women’s sweat are modulated by the gender differences in bacterial compositions [34]. We found significantly more male subjects in the *Prevotella* community class before statistical correction, although this becomes insignificant after correcting for multiple comparisons. Human microbial communities interplay with both environmental and host factors, so the community class pattern may result from the combined influence of both endogenous and exogenous factors.

The habitat-specific community classes for subjects are dynamic. This includes differences based on age [35], point in menstrual cycle [36,37], changes in health states and other lifetime events. It has been reported that bacterial abundance can vary over short periods [38]. We observed that community class stability is habitat-dependent, with the conversion between community classes being more common in type II habitats. It is noteworthy that for those habitats with relatively well-defined classes (skin, vagina, anterior nares and stool), switching mainly occurs in minor community classes whereas dominant community classes maintain dominance over time. The fundamental mechanism of conversion between community classes is not known. Short-term diet can change the microbial composition and abundance, but has not been shown to lead to the replacement of enterotypes [12]. Delimitation of the genetic demographic, environmental, behavioral and nutritional factors that influence community classes in humans is challenging. Animal experiments in a well-controlled setting will be an attractive approach to address to what extent the above factors or a combination of multiple factors contribute to the formation of and changes between community classes. This information will provide significant value in how to alter the human microbiome to prevent or treat disease in the future.

## Conclusion

We identified 2 to 6 community classes for each of the 18 habitats from the HMP healthy cohort by clustering augmented by manual inspection. These community classes are associated with a number of host factors, including gender, race, age and geography, suggesting that the identification of the community classes is non-random. The dynamics of the community classes over a year-long interval underscores the complex interplay of our microbiota with the internal and external environment.

## Materials and methods

### Ethics statement

Subjects provided written informed consent for screening, enrollment and specimen collection. The protocol entitled 'HMP-07-001 Human Microbiome Project - Core Microbiome Sample Protocol A' was reviewed and approved by institutional review board at Washington University in St Louis, IRB ID#: 201105198 (previously 08-0754) and Baylor College of Medicine, IRB ID#: H-22895. The data were analyzed without personal identifiers. Research was conducted according to the principles expressed in the Declaration of Helsinki.

### Sample collection

Specimens were collected by teams at the Baylor College of Medicine and Washington University in St Louis [32]. In total, 236 healthy adults were included in this analysis. Fifteen habitats comprising anterior nares, skin (left and right retroauricular crease, left and right antecubital fossa), oral (hard palate, keratinized gingiva, buccal mucosa, subgingival plaque, supragingival plaque, saliva, tongue dorsum, palatine tonsil and throat) and stool were sampled from all subjects. Female subjects were sampled at three extra sites: vaginal introitus, posterior fornix and mid-vagina. For longitudinal studies, a set of samples from each habitat was collected at two time points (visit one and visit two) separated by 30 to 359 days.

### DNA sequencing, quality control and taxonomic classification

To analyze the 16S rRNA gene, the V3-5 region of the 16S RNA gene was sequenced on the Roche-454 platform to define the composition of the bacterial community. Sample preparation, DNA isolation, sequencing, and data processing were performed following the standardized protocols developed by the HMP consortium [39]. In brief, data processing allowed one mismatch in the barcode and up to two mismatches in primer. The minimal acceptable sequence length was 200 bp.

This dataset is the July 2010 16S rRNA gene sequencing data freeze, 7,518 SRA runs, the Human Microbiome Project 16S rRNA Clinical Production Phase I, available from NCBI at [40], and from the HMP Data Analysis and Coordinating Center at [41].

These sequences were subsequently processed as follows. Chimeric sequences were filtered out by Chimera-Slayer [42]. Average qual 25 was used as the minimal quality score to remove low quality reads. Qualifying sequences were further classified by the Ribosomal Database Project Naive Bayesian Classifier version 2.2 using training set 6 [43] from phylum to genus levels. Taxa assigned <0.5 confidence were reassigned to the next higher taxonomic level in which the classification threshold was >0.5.

Shotgun sequences from posterior fornix, tongue dorsum, supragingival plaque, anterior nares, stool and buccal mucosa were used to confirm the community classes identified by 16S rRNA gene sequencing. Shotgun data were processed by the HMP consortium [39], resulting in measurement of depth and breadth of microbiota based on the reference database [44]. The WGS sequences can be downloaded from [45] and at NCBI at [46].

### Identification of community classes

To cluster subjects with similar bacterial composition in sets of metagenomic samples, we followed the following statistical recipe. First, Ribosomal Database Project data are organized in a matrix format with rows being the subjects, columns being the genera, and entries in the table being the number of reads for that subject by genus combination. These read counts are scaled by dividing the number of reads belonging to that genus by the average copy number of 16S rRNA genes for species belonging to that genus [47]. This was done to avoid overcounting genera with high gene copy numbers. The scaled counts were then transformed to percentages by dividing each count by the total number of scaled counts for that subject. Second, the proximity matrix used for the cluster analysis is built using the Bray-Curtis dissimilarity measure as a pair-wise distance between the genera composition of subjects. The Bray-Curtis dissimilarity measure, *d*(*i*, *j*), quantifies the dissimilarity in species composition between samples *i* and *j*, based on the taxa abundances at each sample, and is defined as:

$$d\left(i,j\right)=\frac{\sum_{k=1}^{n}\left|y_{i,k}-y_{j,k}\right|}{\sum_{k=1}^{n}\left(y_{i,k}+y_{j,k}\right)},$$

where *y*_*i*,*k*_ and *y*_*j*,*k*_ are the abundances (in our case proportions) of genus *k* in samples *i* and *j*, respectively, and *n* is total number of distinct genera present in both samples. The measure *d*(*i*, *j*) ranges between 0 and 1, where 0 means the two samples share all the genera in similar abundances, and 1 means the two subjects do not share any genera at all. This metric was chosen because it is commonly used in ecology because of its robust monotonic and linear relationship with ecological distance [48]. Third, the complete linkage criterion was used to form an agglomerative hierarchical clustering and dendrogram tree for identifying clusters [6]. The complete linkage criterion is a method to calculate distances between two clusters, which is defined as the distance between their most dissimilar members. While there are several criteria for agglomerative clustering, we choose this algorithm because it tends to produce compact clusters. Fourth, to determine the optimal number of clusters within a dendrogram, we used the Silhouette method [49]. The silhouette width *s*(*i*) for each observation j is defined as:

$$s\left(i\right):=\left(b\left(i\right)-a\left(i\right)\right)/\text{max}\left(\;a\left(i\right),b\left(i\right)\;\right)$$

where *a*(*i*) is the average dissimilarity between *i* and all other points of the cluster to which *i* belongs and *b*(*i*), the dissimilarity between *i* and its nearest cluster to which it does not belong. Observations with a large *s*(*i*) (almost 1) are very well clustered, a small *s*(*i*) (around 0) means that the observation lies between two clusters, and observations with a negative *s*(*i*) are probably placed in the wrong cluster [50].

Averaged *s*(*i*) for all the members of the clusters was used to assess the overall cluster quality. The number of clusters that yield the highest silhouette value was chosen to be the optimal number of clusters. Lastly, clusters with similar average silhouette values (<0.02 difference) were manually inspected. We have followed two general rules in this process: (a) within each cluster, silhouette values for the majority of the subjects were high - this ensures the subjects with high similarity are grouped together and avoid heterogeneity within a cluster; (b) clusters with fewer than two samples were removed and cluster analysis was redone with the same procedure. This ensures the clusters we identified are sufficiently representative within a population.

We also performed fuzzy k-means clustering [50], using the same data matrix and dissimilarity measurement as described in hierarchical clustering.

### Principal coordinate analysis (PCoA)

To illustrate the community classes identified in the human habitats, we performed PCoA analysis with the ade4 packages [51] in R. This starts with the Bray-Curtis dissimilarity matrix generated in the clustering analysis and assigns for each sample a location in a two-dimensional space. S class function was used to add additional variables into the graphs as indicated by the community classes with different colors. Cellipse indicating the inertia ellipse size was set to 1.5.

### Indicator genera

Indicator values were calculated using the Dufrene-Legendre Indicator Species approach from the labdsv [25] package in R. The sample frequency (f) and relative average abundance (a) of each genus were calculated as follows:

p_{i,j} = presence/absence (1/0) of species i in sample j;

x_{i,j} = abundance of species i in sample j;

n_c = number of samples in cluster c; ? for cluster c in set K;

f_{i,c} = {∑_{j \in c} p_{i,j} \over n_c}

a_{i,c} = {(∑_{j \in c} x_{i,j}) / n_c \over ∑_{k = 1}^K ((∑_[j 48] x_{i,j}) / n_k)}

d_{i,c} = f_{i,c} \times a_{i,c}

In this analysis, indicator genera were chosen based on: (1) indicator *P*-value <0.01; (2) genera present in at least 50% of subjects in either cluster.

### Quantifying the agreement of clusters between habitats and between visits

The ARI was recommended for measurement of the agreement between two partitions in the clustering analysis after comparing many different indices [52]. The ARI is derived from the Rand Index and is the corrected-for-chance version of this index. It was computed by the fossil package in R [53]. Detailed information can be found in [54]. We used the ARI to compare the cluster similarity between different habitats. Clustering was performed as described above using the samples present in both compared habitats. To evaluate the stability of community class over time, clustering used all data from sampling times 1 and 2.

### Measurement of Shannon diversity

The Shannon index was used to calculate alpha diversity. The samples were first rarified to 1,000 reads by the rarefy function in vegan [55] to prevent the bias caused by different read depth. Shannon diversity was calculated using the BiodiversityR package [56] as described below:

$$H'=-\sum_{i=1}^{S}\left(p_{i}\;\ln\;p_{i}\right)$$

where *S* is the number of species, and *p*_*i*_ is the relative abundance of each species, calculated as the proportion of individuals of a given species to the total number of individuals in the community.

### Association of demographic factors with community classes and single taxa

Demographic factors were mapped to the dendrogram from the hierarchical clustering [57]. The distribution of different of geographical locations (St Louis, Houston), gender (male, female), race and ethnicity (Hispanic/Latino/Spanish, not Hispanic/Latino/Spanish) and BMI (BMI <25, 25 ≤ BMI < 30, BMI ≥30) between community classes were assessed using Fisher’s exact test. The ANOVA test was used when the data were continuous (age). Association of single taxa with gender was assessed by Mann-Whitney-Wilcoxon test. *P*-values from multiple comparisons were corrected using the Bonferroni method. *P*-values <0.05 after correction were considered as significant.

### Data access

The 16S rRNA gene sequences used are the July 2010 16S rRNA gene sequencing data freeze, 7,518 SRA runs, the Human Microbiome Project 16S rRNA Clinical Production Phase I. It is available from NCBI at [40], and from the HMP Data Analysis and Coordinating Center at [41]. The WGS sequences can be downloaded from [45] and at NCBI at [46]. Metadata were downloaded from dbGAP (study accession phs000228.v2.p1) [58].

## Abbreviations

ARI: Adjusted Rand Index; BMI: body mass index; HMP: Human Microbiome Project; PCoA: principal coordinate analysis; WGS: whole genome shotgun.

## Competing interests

The authors declare that they have no competing interests.

## Authors’ contributions

YZ, KAM, HG, PSLA, WDS, GMW, and ES: analysis and interpretation of data. YZ, KAM, HG, PSLA, WDS, MM, GMW, and ES: study design and concept. KMW: dbGap metadata processing. JCM, KK, and MM: WGS data processing. YZ and GMW: drafting the paper. All authors read and approved the final manuscript.

## Supplementary Material

Additional file 1 Tables S1 to S3 and the figure legends for Figures S1 to S8. Click here for file

Additional file 2 Figures S1 to S8. Click here for file
