# Supplementary material for: Exploration of bacterial community classes in major human habitats
Source: Genome Biol. 2014 May 7;15(5):R66. doi: 10.1186/gb-2014-15-5-r66 (PMC4073010; doi:10.1186/gb-2014-15-5-r66)
Supplement: Additional file 1 — Tables S1 to S3 and the figure legends for Figures S1 to S8. [file gb-2014-15-5-r66-S1.docx]

**Additional files**

The following additional data are available with the online version of this paper.

**File name: Additional file 1**

File format: Table

**Table S1. Silhouette values from hierarchical clustering (Hclust) and k-means clustering for**

**models with from 2 to 9 clusters in 16s rRNA gene data.**

| Hierachical clustering | No of clusters | | | | | | | |
| --- | --- | --- | --- | --- | --- | --- | --- | --- |
| Habitats | 2 | 3 | 4 | 5 | 6 | 7 | 8 | 9 |
| Anterior nares | 0.18 | 0.21 | 0.24 | 0.19 | 0.20 | 0.20 | 0.17 | 0.17 |
| Keratinized gingiva | 0.32 | 0.19 | 0.16 | 0.16 | 0.17 | 0.17 | 0.17 | 0.16 |
| Buccal mucosa | 0.28 | 0.22 | 0.22 | 0.19 | 0.17 | 0.17 | 0.18 | 0.17 |
| Hard palate | 0.25 | 0.16 | 0.13 | 0.13 | 0.13 | 0.13 | 0.13 | 0.13 |
| Left antecubital fossa | 0.30 | 0.29 | 0.30 | 0.30 | 0.30 | 0.29 | 0.30 | 0.29 |
| Left retroauricular crease | 0.62 | 0.60 | 0.51 | 0.49 | 0.49 | 0.45 | 0.45 | 0.37 |
| Mid vagina | 0.78 | 0.76 | 0.76 | 0.72 | 0.69 | 0.68 | 0.69 | 0.68 |
| Palatine tonsils | 0.17 | 0.15 | 0.13 | 0.13 | 0.12 | 0.11 | 0.11 | 0.11 |
| Posterior fornix | 0.84 | 0.86 | 0.86 | 0.86 | 0.86 | 0.84 | 0.84 | 0.84 |
| Right antecubital fossa | 0.32 | 0.35 | 0.35 | 0.36 | 0.37 | 0.35 | 0.34 | 0.34 |
| Right retroauricular crease | 0.52 | 0.52 | 0.51 | 0.51 | 0.52 | 0.52 | 0.51 | 0.51 |
| Saliva | 0.14 | 0.13 | 0.09 | 0.09 | 0.09 | 0.08 | 0.08 | 0.07 |
| Stool | 0.21 | 0.25 | 0.20 | 0.21 | 0.19 | 0.19 | 0.17 | 0.17 |
| Subgingival plaque | 0.21 | 0.11 | 0.10 | 0.09 | 0.11 | 0.11 | 0.12 | 0.12 |
| Supragingival plaque | 0.15 | 0.12 | 0.07 | 0.08 | 0.08 | 0.09 | 0.07 | 0.08 |
| Throat | 0.19 | 0.19 | 0.16 | 0.16 | 0.15 | 0.14 | 0.14 | 0.11 |
| Tongue dorsum | 0.13 | 0.18 | 0.14 | 0.15 | 0.11 | 0.10 | 0.10 | 0.08 |
| Vaginal introitus | 0.66 | 0.64 | 0.64 | 0.63 | 0.61 | 0.56 | 0.55 | 0.55 |

| K-means clustering | No of clusters | | | | | | | |
| --- | --- | --- | --- | --- | --- | --- | --- | --- |
| Habitats | 2 | 3 | 4 | 5 | 6 | 7 | 8 | 9 |
| Anterior nares | 0.22 | 0.22 | 0.25 | 0.21 | 0.21 | 0.22 | 0.17 | 0.18 |
| Keratinized gingiva | 0.31 | 0.22 | 0.19 | 0.19 | 0.18 | 0.17 | 0.17 | 0.16 |
| Buccal mucosa | 0.25 | 0.19 | 0.13 | 0.14 | 0.13 | 0.13 | 0.13 | 0.13 |
| Hard palate | 0.22 | 0.22 | 0.19 | 0.16 | 0.13 | 0.13 | 0.12 | 0.12 |
| Left antecubital fossa | 0.23 | 0.25 | 0.27 | 0.26 | 0.20 | 0.20 | 0.14 | 0.21 |
| Left retroauricular crease | 0.54 | 0.45 | 0.32 | 0.32 | 0.34 | 0.28 | 0.28 | 0.29 |
| Mid vagina | 0.71 | 0.68 | 0.51 | 0.31 | 0.31 | 0.33 | 0.34 | 0.35 |
| Palatine tonsils | 0.19 | 0.17 | 0.17 | 0.15 | 0.14 | 0.14 | 0.13 | 0.13 |
| Posterior fornix | 0.86 | 0.86 | 0.65 | 0.35 | 0.35 | 0.36 | 0.40 | 0.37 |
| Right antecubital fossa | 0.30 | 0.16 | 0.19 | 0.16 | 0.12 | 0.19 | 0.15 | 0.17 |
| Right retroauricular crease | 0.51 | 0.35 | 0.38 | 0.32 | 0.29 | 0.29 | 0.29 | 0.30 |
| Saliva | 0.17 | 0.15 | 0.12 | 0.11 | 0.12 | 0.12 | 0.12 | 0.11 |
| Stool | 0.25 | 0.17 | 0.18 | 0.15 | 0.14 | 0.14 | 0.12 | 0.11 |
| Subgingival plaque | 0.21 | 0.15 | 0.11 | 0.11 | 0.12 | 0.13 | 0.12 | 0.12 |
| Supragingival plaque | 0.20 | 0.18 | 0.13 | 0.12 | 0.10 | 0.11 | 0.11 | 0.10 |
| Throat | 0.20 | 0.21 | 0.16 | 0.15 | 0.14 | 0.13 | 0.13 | 0.13 |
| Tongue dorsum | 0.25 | 0.22 | 0.19 | 0.15 | 0.15 | 0.14 | 0.13 | 0.13 |
| Vaginal introitus | 0.62 | 0.42 | 0.36 | 0.40 | 0.41 | 0.34 | 0.30 | 0.33 |

**Table S2. Cluster similarity between different clustering approaches.**

Single linkage tends to produce a very skewed hierarchy therefore is not very useful to summarize the data. Cluster similarity estimated by adjusted rand index (ARI) showed clusters generated from complete linkage of hierarchical clustering is more similar to K-means clustering, while average linkage is more dissimilar to complete linkage and K-means clustering.

|  | complete vs average | complete vs k-means | average vs k-means |
| --- | --- | --- | --- |
| Anterior nares | 0.01 | 0.19 | 0 |
| Keratinized gingiva | 0.01 | 0.57 | 0 |
| Buccal mucosa | 0.08 | 0.11 | 0 |
| Hard palate | 0.31 | 0.01 | 0 |
| Left antecubital fossa | 0.83 | 0.02 | 0.03 |
| Left retroauricular crease | 0.69 | 0.28 | 0.47 |
| Mid vagina | 1 | 0.56 | 0.56 |
| Palatine tonsils | 0.1 | 0.24 | 0.01 |
| Posterior fornix | 0.72 | 0.6 | 0.85 |
| Right antecubital fossa | 0.62 | 0.08 | 0.03 |
| Right retroauricular crease | 0.12 | 0.34 | 0.03 |
| Saliva | 0.07 | 0.35 | 0 |
| Stool | 0.03 | 0.28 | 0 |
| Subgingival plaque | 0.01 | 0.49 | 0 |
| Supragingival plaque | 0 | 0.49 | 0 |
| Throat | 0 | 0.75 | 0 |
| Tongue dorsum | 0.18 | 0.24 | 0.76 |
| Vaginal introitus | 1 | 0.64 | 0.64 |

**Table S3.** **Indicator genera in different habitats.** The frequency (freq) and the relative abundance (relabu) of each indicator genera in different groups (g) are listed in columns. pval refers to the probability of obtaining as high an indicator values as observed over the specified iterations. The table only includes the genera with pval less than or equal 0.01.

**Left Retroauricular crease** (g1- Staphylococcus group; g2- Propionibacterium group; g3- Corynebacterium group)

|  | g1_freq | g2_freq | g3_freq | g1_relabu | g2_relabu | G3_relabu | pval |
| --- | --- | --- | --- | --- | --- | --- | --- |
| Corynebacterium | 1.00 | 0.91 | 1.00 | 0.20 | 0.05 | 0.75 | 0.00 |
| Propionibacterium | 1.00 | 1.00 | 1.00 | 0.17 | 0.76 | 0.08 | 0.00 |
| Staphylococcus | 1.00 | 1.00 | 1.00 | 0.64 | 0.12 | 0.24 | 0.00 |
| Lactobacillus | 0.57 | 0.24 | 0.17 | 0.90 | 0.09 | 0.01 | 0.01 |
| Kingella | 0.50 | 0.23 | 0.67 | 0.14 | 0.04 | 0.83 | 0.01 |
| Pelomonas | 0.21 | 0.27 | 0.83 | 0.05 | 0.04 | 0.91 | 0.00 |
| Bifidobacterium | 0.07 | 0.07 | 0.83 | 0.04 | 0.05 | 0.91 | 0.00 |
| Massilia | 0.14 | 0.06 | 0.50 | 0.01 | 0.01 | 0.98 | 0.00 |
| Selenomonas | 0.07 | 0.08 | 0.50 | 0.10 | 0.13 | 0.77 | 0.00 |
| Kluyvera | 0.07 | 0.10 | 0.50 | 0.03 | 0.02 | 0.95 | 0.00 |
| Comamonadaceae_Unclassified | 0.00 | 0.08 | 0.50 | 0.00 | 0.25 | 0.75 | 0.00 |
| Shuttleworthia | 0.21 | 0.13 | 0.50 | 0.08 | 0.05 | 0.86 | 0.01 |
| Providencia | 0.07 | 0.13 | 0.67 | 0.18 | 0.19 | 0.63 | 0.01 |
| Mitsuokella | 0.14 | 0.10 | 0.50 | 0.19 | 0.05 | 0.75 | 0.01 |

**Right Retroauricular crease** (g1- Staphylococcus group; g2- Propionibacterium group; g3- Pelomonas group; g4- Neisseriaceae group g5-Anaerococcus; g6- Corynebacterium group )

|  | g1_freq | g2_freq | g3_freq | g4_freq | g5_freq | g6_freq | g1_relabu | g2_relabu | g3_relabu | g4_relabu | g5_relabu | g6_relabu | pval |
| --- | --- | --- | --- | --- | --- | --- | --- | --- | --- | --- | --- | --- | --- |
| Propionibacterium | 1.00 | 1.00 | 1.00 | 1.00 | 1.00 | 1.00 | 0.08 | 0.44 | 0.13 | 0.14 | 0.14 | 0.07 | 0.00 |
| Staphylococcus | 1.00 | 1.00 | 1.00 | 1.00 | 1.00 | 1.00 | 0.57 | 0.10 | 0.05 | 0.06 | 0.09 | 0.13 | 0.00 |
| Anaerococcus | 0.63 | 0.56 | 1.00 | 0.25 | 1.00 | 0.25 | 0.11 | 0.06 | 0.01 | 0.03 | 0.79 | 0.00 | 0.00 |
| Corynebacterium | 0.95 | 0.91 | 0.75 | 0.75 | 1.00 | 1.00 | 0.04 | 0.05 | 0.03 | 0.20 | 0.07 | 0.61 | 0.01 |
| Fusobacterium | 0.47 | 0.39 | 1.00 | 0.25 | 0.75 | 0.50 | 0.02 | 0.02 | 0.85 | 0.06 | 0.04 | 0.01 | 0.00 |
| Ralstonia | 0.26 | 0.21 | 1.00 | 0.50 | 0.25 | 0.25 | 0.01 | 0.09 | 0.88 | 0.01 | 0.00 | 0.01 | 0.00 |
| Neisseriaceae_Unclassified | 0.21 | 0.21 | 0.75 | 1.00 | 0.00 | 0.50 | 0.00 | 0.01 | 0.01 | 0.96 | 0.00 | 0.02 | 0.00 |
| Ruminococcaceae_Unclassified | 0.16 | 0.13 | 1.00 | 0.50 | 0.25 | 0.50 | 0.01 | 0.03 | 0.84 | 0.06 | 0.03 | 0.03 | 0.00 |
| Kluyvera | 0.16 | 0.06 | 0.75 | 0.25 | 0.25 | 0.00 | 0.02 | 0.03 | 0.85 | 0.08 | 0.02 | 0.00 | 0.00 |
| Providencia | 0.16 | 0.08 | 0.75 | 0.25 | 0.00 | 0.00 | 0.01 | 0.01 | 0.93 | 0.05 | 0.00 | 0.00 | 0.00 |
| Shuttleworthia | 0.11 | 0.13 | 1.00 | 0.50 | 0.00 | 0.00 | 0.00 | 0.00 | 0.97 | 0.03 | 0.00 | 0.00 | 0.00 |
| Dorea | 0.05 | 0.15 | 1.00 | 0.50 | 0.75 | 0.00 | 0.01 | 0.01 | 0.82 | 0.05 | 0.10 | 0.00 | 0.00 |
| Simonsiella | 0.05 | 0.06 | 0.50 | 1.00 | 0.00 | 0.50 | 0.00 | 0.03 | 0.06 | 0.87 | 0.00 | 0.04 | 0.00 |
| Kingella | 0.37 | 0.23 | 1.00 | 0.50 | 0.25 | 0.00 | 0.03 | 0.03 | 0.81 | 0.07 | 0.07 | 0.00 | 0.00 |
| Pelomonas | 0.32 | 0.34 | 1.00 | 0.50 | 0.25 | 0.25 | 0.01 | 0.01 | 0.95 | 0.00 | 0.00 | 0.02 | 0.00 |
| Sphingobium | 0.16 | 0.17 | 0.75 | 0.00 | 0.25 | 0.25 | 0.03 | 0.01 | 0.94 | 0.00 | 0.01 | 0.02 | 0.00 |
| Mitsuokella | 0.26 | 0.14 | 0.75 | 0.25 | 0.00 | 0.00 | 0.05 | 0.03 | 0.86 | 0.06 | 0.00 | 0.00 | 0.00 |
| Collinsella | 0.00 | 0.05 | 0.50 | 0.00 | 0.00 | 0.00 | 0.00 | 0.01 | 0.99 | 0.00 | 0.00 | 0.00 | 0.00 |
| Enterobacter | 0.16 | 0.10 | 0.75 | 0.25 | 0.25 | 0.00 | 0.04 | 0.04 | 0.75 | 0.05 | 0.12 | 0.00 | 0.01 |
| Burkholderia | 0.11 | 0.01 | 0.50 | 0.00 | 0.00 | 0.00 | 0.06 | 0.01 | 0.93 | 0.00 | 0.00 | 0.00 | 0.01 |
| Enterobacteriaceae_Unclassified | 0.11 | 0.14 | 1.00 | 0.75 | 1.00 | 0.00 | 0.01 | 0.03 | 0.54 | 0.16 | 0.27 | 0.00 | 0.01 |
| Arthrobacter | 0.05 | 0.03 | 0.50 | 0.00 | 0.25 | 0.00 | 0.03 | 0.00 | 0.95 | 0.00 | 0.02 | 0.00 | 0.01 |
| Stenoxybacter | 0.05 | 0.12 | 0.00 | 1.00 | 0.00 | 0.50 | 0.00 | 0.00 | 0.00 | 0.98 | 0.00 | 0.01 | 0.00 |
| Corynebacteriaceae_Unclassified | 0.00 | 0.01 | 0.00 | 0.50 | 0.00 | 0.00 | 0.00 | 0.02 | 0.00 | 0.98 | 0.00 | 0.00 | 0.01 |
| Peptoniphilus | 0.11 | 0.23 | 0.00 | 0.25 | 1.00 | 0.25 | 0.01 | 0.01 | 0.00 | 0.06 | 0.92 | 0.00 | 0.00 |
| Wautersiella | 0.00 | 0.03 | 0.00 | 0.00 | 0.50 | 0.00 | 0.00 | 0.04 | 0.00 | 0.00 | 0.96 | 0.00 | 0.01 |

**Mid Vagina** (g1- Lactobacillus group; g2- Anerobic group)

|  | g1_freq | g2_freq | g1_relabu | g2_relabu | pval |
| --- | --- | --- | --- | --- | --- |
| Lactobacillus | 1.00 | 1.00 | 0.95 | 0.05 | 0.00 |
| Lactobacillaceae_Unclassified | 0.72 | 0.00 | 1.00 | 0.00 | 0.00 |
| Prevotella | 0.48 | 1.00 | 0.08 | 0.92 | 0.00 |
| Dialister | 0.46 | 1.00 | 0.15 | 0.85 | 0.00 |
| Clostridiales_Unclassified | 0.37 | 1.00 | 0.02 | 0.98 | 0.00 |
| Gardnerella | 0.32 | 1.00 | 0.09 | 0.91 | 0.00 |
| Veillonellaceae_Unclassified | 0.21 | 1.00 | 0.09 | 0.91 | 0.00 |
| Atopobium | 0.17 | 0.86 | 0.02 | 0.98 | 0.00 |
| Lachnospiraceae_Unclassified | 0.14 | 0.71 | 0.01 | 0.99 | 0.00 |
| Aerococcus | 0.06 | 0.86 | 0.10 | 0.90 | 0.00 |
| Ruminococcaceae_Unclassified | 0.06 | 0.86 | 0.04 | 0.96 | 0.00 |
| Bifidobacteriaceae_Unclassified | 0.05 | 0.57 | 0.04 | 0.96 | 0.00 |
| Moryella | 0.04 | 0.57 | 0.08 | 0.92 | 0.00 |
| Anaeroglobus | 0.04 | 0.57 | 0.17 | 0.83 | 0.00 |
| Megasphaera | 0.04 | 0.86 | 0.00 | 1.00 | 0.00 |
| Unclassified_Bacteria | 0.04 | 0.57 | 0.06 | 0.94 | 0.00 |
| Acetivibrio | 0.02 | 0.86 | 0.00 | 1.00 | 0.00 |
| Coriobacteriaceae_Unclassified | 0.01 | 0.71 | 0.00 | 1.00 | 0.00 |
| Enterorhabdus | 0.00 | 0.71 | 0.00 | 1.00 | 0.00 |
| Parvimonas | 0.00 | 0.86 | 0.00 | 1.00 | 0.00 |
| Sneathia | 0.00 | 0.71 | 0.00 | 1.00 | 0.00 |
| Howardella | 0.12 | 0.57 | 0.24 | 0.76 | 0.01 |

**Vaginal introitus** (g1- Lactobacillus group; g2- Anerobic group)

|  | cc1_freq | cc2_freq | cc1_relabu | cc2_relabu | pval |
| --- | --- | --- | --- | --- | --- |
| Lactobacillus | 1.00 | 1.00 | 0.87 | 0.13 | 0.00 |
| Lactobacillales_Unclassified | 0.88 | 0.46 | 0.76 | 0.24 | 0.00 |
| Prevotella | 0.66 | 1.00 | 0.07 | 0.93 | 0.00 |
| Peptoniphilus | 0.60 | 0.92 | 0.16 | 0.84 | 0.00 |
| Dialister | 0.55 | 1.00 | 0.06 | 0.94 | 0.00 |
| Lactobacillaceae_Unclassified | 0.76 | 0.38 | 0.79 | 0.21 | 0.00 |
| Clostridiales_Unclassified | 0.45 | 0.85 | 0.10 | 0.90 | 0.00 |
| Gardnerella | 0.28 | 0.85 | 0.14 | 0.86 | 0.00 |
| Veillonellaceae_Unclassified | 0.22 | 0.69 | 0.21 | 0.79 | 0.00 |
| Atopobium | 0.19 | 1.00 | 0.05 | 0.95 | 0.00 |
| Campylobacter | 0.13 | 0.62 | 0.03 | 0.97 | 0.00 |
| Megasphaera | 0.10 | 0.54 | 0.00 | 1.00 | 0.00 |
| Anaeroglobus | 0.03 | 0.54 | 0.01 | 0.99 | 0.00 |
| Coriobacteriaceae_Unclassified | 0.01 | 0.54 | 0.01 | 0.99 | 0.00 |
| Howardella | 0.18 | 0.62 | 0.11 | 0.89 | 0.00 |
| Aerococcus | 0.07 | 0.54 | 0.21 | 0.79 | 0.00 |

**Posterior fornix** (g1- Lactobacillus group; g2- Prevotella group,g3- Gardnerella)

|  | g1_freq | g2_freq | g3_freq | g1_relabu | g2_relabu | g3_relabu | pval |
| --- | --- | --- | --- | --- | --- | --- | --- |
| Lactobacillus | 1 | 1 | 1 | 0.91 | 0.01 | 0.08 | 0.001 |
| Lactobacillales_Unclassified | 0.91 | 0 | 0.67 | 0.88 | 0 | 0.12 | 0.001 |
| Lactobacillaceae_Unclassified | 0.76 | 0 | 0.33 | 0.93 | 0 | 0.07 | 0.001 |
| Veillonellaceae_Unclassified | 0.04 | 1 | 0 | 0.01 | 0.99 | 0 | 0.001 |
| Ruminococcaceae_Unclassified | 0.03 | 0.8 | 0 | 0.01 | 0.99 | 0 | 0.001 |
| Parvimonas | 0.01 | 0.8 | 0 | 0 | 1 | 0 | 0.001 |
| Moryella | 0.01 | 0.8 | 0 | 0.01 | 0.99 | 0 | 0.001 |
| Megasphaera | 0.01 | 1 | 0 | 0 | 1 | 0 | 0.001 |
| Coriobacteriaceae_Unclassified | 0 | 0.8 | 0 | 0 | 1 | 0 | 0.001 |
| Acetivibrio | 0 | 1 | 0 | 0 | 1 | 0 | 0.001 |
| Sneathia | 0 | 0.8 | 0 | 0 | 1 | 0 | 0.001 |
| Anaeroglobus | 0 | 0.6 | 0 | 0 | 1 | 0 | 0.002 |
| Enterorhabdus | 0 | 0.6 | 0 | 0 | 1 | 0 | 0.003 |
| Clostridiales_Unclassified | 0.23 | 0.8 | 0 | 0.01 | 0.99 | 0 | 0.005 |
| Lachnospiraceae_Unclassified | 0.08 | 0.6 | 0 | 0 | 1 | 0 | 0.005 |
| Prevotella | 0.28 | 1 | 0.33 | 0.02 | 0.9 | 0.09 | 0.006 |
| Peptoniphilus | 0.19 | 1 | 0.33 | 0.07 | 0.79 | 0.14 | 0.006 |
| Gardnerella | 0.24 | 0.8 | 1 | 0.02 | 0.17 | 0.81 | 0.008 |
| Bifidobacterium | 0.05 | 0.4 | 0.67 | 0.02 | 0 | 0.97 | 0.002 |
| Anaerococcus | 0.16 | 0.4 | 0.67 | 0.02 | 0.04 | 0.94 | 0.009 |

| **genus** | **g1_freq** | **g2_freq** | **g3_freq** | **g4_freq** | **g1_relabu** | **g2_relabu** | **g3_relabu** | **g4_relabu** | **pval** |
| --- | --- | --- | --- | --- | --- | --- | --- | --- | --- |
| **Corynebacterium** | 1.00 | 0.95 | 1.00 | 1.00 | 0.52 | 0.16 | 0.25 | 0.07 | 0.001 |
| **Propionibacterium** | 1.00 | 1.00 | 1.00 | 1.00 | 0.24 | 0.47 | 0.08 | 0.21 | 0.001 |
| **Staphylococcus** | 1.00 | 1.00 | 1.00 | 1.00 | 0.16 | 0.11 | 0.09 | 0.64 | 0.001 |
| **Staphylococcaceae_Unclassified** | 0.72 | 0.61 | 0.63 | 1.00 | 0.23 | 0.17 | 0.12 | 0.47 | 0.002 |
| **Moraxella** | 0.11 | 0.23 | 1.00 | 0.00 | 0.01 | 0.05 | 0.94 | 0.00 | 0.001 |

**Anterior Nares** (g1- Corynebacterium group; g2- Propionibacterium group; g3- Moraxella group; g4- Staphylococcus group)

**Left Antecubital Fossa** (g1- Propionibacterium group; g2- Sporacetigenium group; g3- Corynebacterium group; g4- Ralstonia group;g5- Staphylococcus group)

|  | g1_freq | | g2_freq | | g3_freq | g4_freq | g5_freq | | g1_relabu | g2_relabu | g3_relabu | g4_relabu | g5_relabu | pval |
| --- | --- | --- | --- | --- | --- | --- | --- | --- | --- | --- | --- | --- | --- | --- |
| Propionibacterium | 1.00 | 1.00 | | 1.00 | | 1.00 | | 1.00 | 0.49 | 0.11 | 0.21 | 0.06 | 0.13 | 0.001 |
| Staphylococcus | 1.00 | 1.00 | | 1.00 | | 1.00 | 1.00 | | 0.05 | 0.01 | 0.05 | 0.07 | 0.83 | 0.001 |
| Gemella | 0.68 | 0.50 | | 0.83 | | 1.00 | 0.33 | | 0.07 | 0.02 | 0.19 | 0.68 | 0.04 | 0.001 |
| Lachnospiraceae_Unclassified | 0.54 | 1.00 | | 0.80 | | 0.71 | 0.50 | | 0.02 | 0.82 | 0.11 | 0.03 | 0.02 | 0.001 |
| Fusobacterium | 0.54 | 0.50 | | 0.90 | | 1.00 | 0.33 | | 0.05 | 0.02 | 0.21 | 0.71 | 0.01 | 0.001 |
| Sphingomonas | 0.54 | 0.00 | | 0.57 | | 1.00 | 0.83 | | 0.06 | 0.00 | 0.13 | 0.69 | 0.11 | 0.001 |
| Coprococcus | 0.68 | 1.00 | | 0.63 | | 0.86 | 0.83 | | 0.03 | 0.70 | 0.10 | 0.12 | 0.04 | 0.005 |
| Pelomonas | 0.43 | 0.50 | | 0.43 | | 1.00 | 0.33 | | 0.04 | 0.01 | 0.04 | 0.89 | 0.02 | 0.001 |
| Enterobacteriaceae_Unclassified | 0.39 | 1.00 | | 0.47 | | 1.00 | 0.17 | | 0.02 | 0.93 | 0.02 | 0.03 | 0.00 | 0.001 |
| Dorea | 0.25 | 1.00 | | 0.43 | | 0.57 | 0.33 | | 0.01 | 0.90 | 0.01 | 0.07 | 0.01 | 0.001 |
| Sporacetigenium | 0.21 | 1.00 | | 0.17 | | 0.00 | 0.17 | | 0.01 | 0.99 | 0.00 | 0.00 | 0.00 | 0.001 |
| Raoultella | 0.14 | 1.00 | | 0.03 | | 0.00 | 0.00 | | 0.02 | 0.96 | 0.01 | 0.00 | 0.00 | 0.001 |
| Citrobacter | 0.11 | 1.00 | | 0.10 | | 0.00 | 0.17 | | 0.01 | 0.99 | 0.00 | 0.00 | 0.00 | 0.001 |
| Dysgonomonas | 0.07 | 1.00 | | 0.00 | | 0.00 | 0.00 | | 0.00 | 1.00 | 0.00 | 0.00 | 0.00 | 0.001 |
| Obesumbacterium | 0.04 | 1.00 | | 0.00 | | 0.00 | 0.00 | | 0.00 | 1.00 | 0.00 | 0.00 | 0.00 | 0.001 |
| Klebsiella | 0.14 | 1.00 | | 0.10 | | 0.29 | 0.00 | | 0.06 | 0.82 | 0.05 | 0.08 | 0.00 | 0.002 |
| Phenylobacterium | 0.18 | 0.50 | | 0.17 | | 0.86 | 0.33 | | 0.02 | 0.02 | 0.08 | 0.85 | 0.03 | 0.006 |
| Yersinia | 0.04 | 1.00 | | 0.03 | | 0.00 | 0.33 | | 0.05 | 0.64 | 0.01 | 0.00 | 0.31 | 0.009 |
| Ralstonia | 0.21 | 0.00 | | 0.23 | | 1.00 | 0.17 | | 0.00 | 0.00 | 0.03 | 0.97 | 0.00 | 0.001 |
| Mitsuokella | 0.18 | 0.00 | | 0.07 | | 0.86 | 0.00 | | 0.04 | 0.00 | 0.03 | 0.93 | 0.00 | 0.003 |
| Kingella | 0.39 | 0.00 | | 0.40 | | 1.00 | 0.00 | | 0.06 | 0.00 | 0.15 | 0.79 | 0.00 | 0.005 |
| Staphylococcaceae_Unclassified | 0.29 | 0.00 | | 0.47 | | 0.57 | 1.00 | | 0.05 | 0.00 | 0.10 | 0.13 | 0.72 | 0.007 |
| Bradyrhizobium | 0.21 | 0.00 | | 0.20 | | 0.86 | 0.17 | | 0.04 | 0.00 | 0.02 | 0.93 | 0.00 | 0.008 |
| Corynebacterium | 1.00 | 1.00 | | 1.00 | | 0.86 | 1.00 | | 0.12 | 0.14 | 0.37 | 0.27 | 0.10 | 0.18 |

**Right Antecubital Fossa** (g1- Streptophyta; g2- Propionibacterium group; g3- Heterogeneous group; g4- Streptococcus group; g5- Staphylococcus group; g6- Corynebacterium group;)

|  | g1_freq | g2_freq | g3_freq | g4_freq | g5_freq | g6_freq | g1_relabu | g2_relabu | g3_relabu | g4_relabu | g5_relabu | g6_relabu | pval |
| --- | --- | --- | --- | --- | --- | --- | --- | --- | --- | --- | --- | --- | --- |
| Corynebacterium | 1.00 | 1.00 | 1.00 | 0.67 | 1.00 | 1.00 | 0.03 | 0.09 | 0.07 | 0.04 | 0.03 | 0.74 | 0.001 |
| Propionibacterium | 1.00 | 1.00 | 1.00 | 1.00 | 1.00 | 1.00 | 0.04 | 0.50 | 0.16 | 0.06 | 0.09 | 0.15 | 0.001 |
| Streptophyta | 1.00 | 0.62 | 0.63 | 0.33 | 0.50 | 1.00 | 0.96 | 0.01 | 0.02 | 0.00 | 0.01 | 0.00 | 0.001 |
| Staphylococcus | 1.00 | 1.00 | 1.00 | 0.67 | 1.00 | 1.00 | 0.01 | 0.04 | 0.02 | 0.08 | 0.76 | 0.09 | 0.001 |
| Chloroplast_Unclassified | 0.67 | 0.02 | 0.00 | 0.00 | 0.00 | 0.00 | 0.99 | 0.01 | 0.00 | 0.00 | 0.00 | 0.00 | 0.002 |
| Myxococcales_Unclassified | 0.67 | 0.02 | 0.00 | 0.00 | 0.00 | 0.00 | 1.00 | 0.00 | 0.00 | 0.00 | 0.00 | 0.00 | 0.002 |
| Proteobacteria_Unclassified | 1.00 | 0.07 | 0.25 | 0.00 | 0.00 | 0.67 | 0.75 | 0.01 | 0.02 | 0.00 | 0.00 | 0.23 | 0.003 |
| Streptococcus | 1.00 | 0.98 | 0.88 | 1.00 | 1.00 | 1.00 | 0.12 | 0.08 | 0.06 | 0.67 | 0.02 | 0.04 | 0.004 |
| Actinomycetales_Unclassified | 0.67 | 0.77 | 0.50 | 0.17 | 0.50 | 1.00 | 0.08 | 0.09 | 0.03 | 0.01 | 0.04 | 0.76 | 0.007 |
| Filifactor | 0.67 | 0.00 | 0.00 | 0.00 | 0.25 | 0.00 | 0.68 | 0.00 | 0.00 | 0.00 | 0.32 | 0.00 | 0.009 |
| Haemophilus | 0.33 | 0.69 | 0.88 | 1.00 | 1.00 | 1.00 | 0.00 | 0.04 | 0.04 | 0.85 | 0.05 | 0.03 | 0.004 |
| Actinobacillus | 0.33 | 0.39 | 0.75 | 1.00 | 0.75 | 0.33 | 0.01 | 0.05 | 0.04 | 0.81 | 0.08 | 0.01 | 0.006 |
| Staphylococcaceae_Unclassified | 0.00 | 0.39 | 0.25 | 0.33 | 1.00 | 0.33 | 0.00 | 0.04 | 0.03 | 0.05 | 0.85 | 0.03 | 0.001 |
| Bacillales_Unclassified | 0.00 | 0.25 | 0.25 | 0.33 | 1.00 | 0.33 | 0.00 | 0.04 | 0.13 | 0.05 | 0.75 | 0.03 | 0.002 |
| Longilinea | 0.00 | 0.02 | 0.00 | 0.00 | 0.00 | 0.67 | 0.00 | 0.03 | 0.00 | 0.00 | 0.00 | 0.97 | 0.003 |
| Corynebacteriaceae_Unclassified | 0.00 | 0.05 | 0.00 | 0.00 | 0.00 | 0.67 | 0.00 | 0.07 | 0.00 | 0.00 | 0.00 | 0.93 | 0.005 |
| Anaerolineaceae_Unclassified | 0.00 | 0.00 | 0.00 | 0.00 | 0.00 | 0.67 | 0.00 | 0.00 | 0.00 | 0.00 | 0.00 | 1.00 | 0.005 |
| Nocardiaceae_Unclassified | 0.00 | 0.08 | 0.13 | 0.00 | 0.00 | 0.67 | 0.00 | 0.07 | 0.07 | 0.00 | 0.00 | 0.85 | 0.01 |

**Keratinized Gingiva (g1-Streptococcus group; g2- Unclassified group)**

| **genus** | **g1_freq** | **g2_freq** | **g1_relabu** | **g2_relabu** | **pval** |
| --- | --- | --- | --- | --- | --- |
| **Streptococcus** | 1.00 | 1.00 | 0.70 | 0.30 | 0.001 |
| **Haemophilus** | 1.00 | 1.00 | 0.66 | 0.34 | 0.001 |
| **Pasteurellaceae_Unclassified** | 1.00 | 1.00 | 0.66 | 0.34 | 0.001 |
| **Veillonella** | 0.99 | 1.00 | 0.25 | 0.75 | 0.001 |
| **Actinobacillus** | 0.99 | 0.98 | 0.72 | 0.28 | 0.001 |
| **Granulicatella** | 0.92 | 1.00 | 0.34 | 0.66 | 0.001 |
| **Fusobacterium** | 0.89 | 1.00 | 0.22 | 0.78 | 0.001 |
| **Prevotella** | 0.84 | 0.97 | 0.30 | 0.70 | 0.001 |
| **Porphyromonas** | 0.75 | 0.97 | 0.27 | 0.73 | 0.001 |
| **Prevotellaceae_Unclassified** | 0.68 | 0.97 | 0.09 | 0.91 | 0.001 |
| **Bacteroidales_Unclassified** | 0.54 | 0.98 | 0.12 | 0.88 | 0.001 |
| **Campylobacter** | 0.51 | 0.71 | 0.27 | 0.73 | 0.002 |
| **Rothia** | 0.54 | 0.42 | 0.82 | 0.18 | 0.009 |
| **Bacteroides** | 0.42 | 0.93 | 0.18 | 0.82 | 0.001 |
| **Unclassified_Bacteria** | 0.24 | 0.51 | 0.29 | 0.71 | 0.001 |
| **Porphyromonadaceae_Unclassified** | 0.17 | 0.69 | 0.15 | 0.85 | 0.001 |
| **Veillonellaceae_Unclassified** | 0.17 | 0.53 | 0.20 | 0.80 | 0.001 |
| **Paraprevotella** | 0.36 | 0.78 | 0.41 | 0.59 | 0.002 |

**Stool** (g1-Bacteroides group; g2-Ruminococcaceae, Clostridiales group; g3-Prevotella group)

| **genus** | **g1_freq** | **g2_freq** | **g3_freq** | **g1_relabu** | **g2_relabu** | **g3_relabu** | **pval** |
| --- | --- | --- | --- | --- | --- | --- | --- |
| **Bacteroides** | 1.00 | 1.00 | 1.00 | 0.58 | 0.20 | 0.22 | 0.001 |
| **Clostridiales_Unclassified** | 0.98 | 1.00 | 1.00 | 0.20 | 0.60 | 0.20 | 0.001 |
| **Ruminococcaceae_Unclassified** | 0.97 | 1.00 | 0.93 | 0.21 | 0.63 | 0.16 | 0.001 |
| **Oscillibacter** | 0.96 | 1.00 | 0.93 | 0.22 | 0.55 | 0.24 | 0.001 |
| **Subdoligranulum** | 0.92 | 1.00 | 0.93 | 0.25 | 0.69 | 0.06 | 0.001 |
| **Alistipes** | 0.85 | 1.00 | 0.80 | 0.25 | 0.56 | 0.19 | 0.001 |
| **Acetivibrio** | 0.72 | 0.95 | 0.93 | 0.24 | 0.61 | 0.15 | 0.001 |
| **Sporobacter** | 0.53 | 0.98 | 0.80 | 0.23 | 0.62 | 0.15 | 0.001 |
| **Anaerotruncus** | 0.68 | 0.92 | 0.67 | 0.27 | 0.56 | 0.17 | 0.003 |
| **Ethanoligenens** | 0.49 | 0.89 | 0.67 | 0.14 | 0.69 | 0.16 | 0.001 |
| **Prevotella** | 0.23 | 0.56 | 1.00 | 0.01 | 0.07 | 0.92 | 0.001 |
| **Bacteroidales_Unclassified** | 0.43 | 0.73 | 0.93 | 0.09 | 0.21 | 0.70 | 0.002 |
| **Veillonellaceae_Unclassified** | 0.30 | 0.80 | 0.47 | 0.16 | 0.55 | 0.29 | 0.002 |
| **Acetanaerobacterium** | 0.48 | 0.83 | 0.60 | 0.11 | 0.68 | 0.22 | 0.003 |
| **Rikenellaceae_Unclassified** | 0.26 | 0.56 | 0.33 | 0.13 | 0.77 | 0.10 | 0.003 |
| **Erysipelotrichaceae_Unclassified** | 0.19 | 0.61 | 0.47 | 0.07 | 0.68 | 0.25 | 0.003 |
| **Firmicutes_Unclassified** | 0.24 | 0.67 | 0.53 | 0.09 | 0.63 | 0.28 | 0.004 |
| **Akkermansia** | 0.23 | 0.55 | 0.13 | 0.12 | 0.86 | 0.02 | 0.004 |
| **Burkholderiales_Unclassified** | 0.16 | 0.21 | 0.60 | 0.08 | 0.05 | 0.87 | 0.001 |
| **Prevotellaceae_Unclassified** | 0.08 | 0.30 | 0.87 | 0.12 | 0.17 | 0.72 | 0.001 |
| **Xylanibacter** | 0.02 | 0.14 | 0.80 | 0.00 | 0.15 | 0.85 | 0.001 |

**Buccal Mucosa** (g1-Streptococcus group; g2- Haemophilus, Fusobacterium and more group)

| **genus** | **g1_freq** | **g2_freq** | **g1_relabu** | **g2_relabu** | **pval** |
| --- | --- | --- | --- | --- | --- |
| **Streptococcus** | 1.00 | 1.00 | 0.69 | 0.31 | 0.001 |
| **Fusobacterium** | 1.00 | 1.00 | 0.32 | 0.68 | 0.001 |
| **Veillonella** | 0.99 | 1.00 | 0.34 | 0.66 | 0.001 |
| **Haemophilus** | 0.99 | 1.00 | 0.36 | 0.64 | 0.001 |
| **Porphyromonas** | 0.97 | 1.00 | 0.30 | 0.70 | 0.001 |
| **Capnocytophaga** | 0.91 | 1.00 | 0.23 | 0.77 | 0.001 |
| **Syntrophococcus** | 0.81 | 0.94 | 0.26 | 0.74 | 0.001 |
| **Lachnospiraceae_Unclassified** | 0.80 | 0.94 | 0.31 | 0.69 | 0.001 |
| **Paraprevotella** | 0.68 | 0.94 | 0.26 | 0.74 | 0.001 |
| **Neisseria** | 0.98 | 1.00 | 0.35 | 0.65 | 0.002 |
| **Leptotrichia** | 0.95 | 1.00 | 0.31 | 0.69 | 0.002 |
| **Pasteurella** | 0.76 | 0.94 | 0.34 | 0.66 | 0.002 |
| **Prevotellaceae_Unclassified** | 0.80 | 1.00 | 0.31 | 0.69 | 0.003 |
| **Oribacterium** | 0.78 | 0.88 | 0.29 | 0.71 | 0.003 |
| **Planobacterium** | 0.78 | 0.94 | 0.37 | 0.63 | 0.004 |
| **Kingella** | 0.75 | 1.00 | 0.33 | 0.67 | 0.004 |
| **Campylobacter** | 0.63 | 0.81 | 0.32 | 0.68 | 0.004 |
| **Prevotella** | 0.97 | 0.97 | 0.35 | 0.65 | 0.009 |
| **Tannerella** | 0.39 | 0.59 | 0.19 | 0.81 | 0.001 |
| **Peptostreptococcus** | 0.35 | 0.59 | 0.22 | 0.78 | 0.001 |
| **Unclassified_Bacteria** | 0.31 | 0.63 | 0.31 | 0.69 | 0.002 |
| **Centipeda** | 0.44 | 0.69 | 0.34 | 0.66 | 0.003 |
| **Alcaligenaceae_Unclassified** | 0.39 | 0.66 | 0.38 | 0.62 | 0.01 |

**Hard Palate** (g1-Streptococcus group; g2- Veillonella, Actinomyces and more group)

| **genus** | **g1_freq** | **g2_freq** | **g1_relabu** | **g2_relabu** | **pval** |
| --- | --- | --- | --- | --- | --- |
| **Prevotella** | 1.00 | 1.00 | 0.28 | 0.72 | 0.001 |
| **Streptococcus** | 1.00 | 1.00 | 0.74 | 0.26 | 0.001 |
| **Actinomyces** | 0.99 | 1.00 | 0.25 | 0.75 | 0.001 |
| **Gemella** | 0.99 | 1.00 | 0.87 | 0.13 | 0.001 |
| **Veillonella** | 0.99 | 1.00 | 0.33 | 0.67 | 0.001 |
| **Megasphaera** | 0.74 | 0.90 | 0.16 | 0.84 | 0.001 |
| **Bacteroidales_Unclassified** | 0.77 | 1.00 | 0.27 | 0.73 | 0.002 |
| **Haemophilus** | 1.00 | 1.00 | 0.67 | 0.33 | 0.003 |
| **Moryella** | 0.91 | 1.00 | 0.22 | 0.78 | 0.003 |
| **Atopobium** | 0.92 | 1.00 | 0.26 | 0.74 | 0.005 |
| **Paraprevotella** | 0.89 | 0.90 | 0.24 | 0.76 | 0.005 |
| **Porphyromonas** | 0.96 | 1.00 | 0.76 | 0.24 | 0.006 |
| **Centipeda** | 0.70 | 0.90 | 0.24 | 0.76 | 0.006 |

**Tongue dorsum** (g1-Actinomyces group; g2- Neisseria group)

| **genus** | **g1_freq** | **g2_freq** | **g1_relabu** | **g2_relabu** | **pval** |
| --- | --- | --- | --- | --- | --- |
| **Actinomyces** | 1.00 | 1.00 | 0.31 | 0.69 | 0.001 |
| **Prevotella** | 1.00 | 1.00 | 0.35 | 0.65 | 0.001 |
| **Gemella** | 1.00 | 0.96 | 0.63 | 0.37 | 0.001 |
| **Streptococcus** | 1.00 | 1.00 | 0.39 | 0.61 | 0.001 |
| **Veillonella** | 1.00 | 1.00 | 0.37 | 0.63 | 0.001 |
| **Fusobacterium** | 1.00 | 0.99 | 0.73 | 0.27 | 0.001 |
| **Neisseria** | 1.00 | 0.94 | 0.86 | 0.15 | 0.001 |
| **Actinobacillus** | 1.00 | 0.92 | 0.72 | 0.28 | 0.001 |
| **Haemophilus** | 1.00 | 0.99 | 0.68 | 0.32 | 0.001 |
| **Pasteurellaceae_Unclassified** | 1.00 | 0.98 | 0.69 | 0.31 | 0.001 |
| **Porphyromonas** | 0.99 | 0.79 | 0.90 | 0.10 | 0.001 |
| **Oribacterium** | 0.99 | 1.00 | 0.38 | 0.62 | 0.001 |
| **Syntrophococcus** | 0.99 | 0.99 | 0.39 | 0.61 | 0.001 |
| **Pasteurella** | 0.96 | 0.63 | 0.73 | 0.27 | 0.001 |
| **Flavobacteriaceae_Unclassified** | 0.95 | 0.58 | 0.78 | 0.22 | 0.001 |
| **Prevotellaceae_Unclassified** | 0.93 | 0.89 | 0.72 | 0.28 | 0.001 |
| **Moryella** | 0.91 | 0.97 | 0.24 | 0.76 | 0.001 |
| **Atopobium** | 0.90 | 1.00 | 0.15 | 0.85 | 0.001 |
| **Peptostreptococcus** | 0.89 | 0.68 | 0.65 | 0.35 | 0.001 |
| **Planobacterium** | 0.88 | 0.46 | 0.82 | 0.18 | 0.001 |
| **Capnocytophaga** | 0.85 | 0.66 | 0.84 | 0.16 | 0.001 |
| **Neisseriaceae_Unclassified** | 0.74 | 0.38 | 0.78 | 0.22 | 0.001 |
| **Campylobacter** | 0.72 | 0.87 | 0.28 | 0.72 | 0.001 |
| **Parvimonas** | 0.71 | 0.37 | 0.82 | 0.18 | 0.001 |
| **Kingella** | 0.65 | 0.32 | 0.77 | 0.23 | 0.001 |
| **Lactobacillales_Unclassified** | 0.59 | 0.88 | 0.36 | 0.64 | 0.001 |
| **Mogibacterium** | 0.55 | 0.79 | 0.31 | 0.69 | 0.001 |
| **Megasphaera** | 0.54 | 0.94 | 0.14 | 0.86 | 0.001 |
| **Peptococcus** | 0.53 | 0.16 | 0.85 | 0.15 | 0.001 |
| **Centipeda** | 0.51 | 0.78 | 0.20 | 0.80 | 0.001 |
| **Leptotrichia** | 1.00 | 0.96 | 0.33 | 0.67 | 0.002 |
| **Bacteroidales_Unclassified** | 0.87 | 0.79 | 0.68 | 0.32 | 0.002 |
| **Filifactor** | 0.80 | 0.63 | 0.65 | 0.35 | 0.002 |
| **Veillonellaceae_Unclassified** | 0.36 | 0.72 | 0.26 | 0.74 | 0.001 |
| **Actinomycetales_Unclassified** | 0.34 | 0.67 | 0.34 | 0.66 | 0.001 |
| **Selenomonas** | 0.33 | 0.59 | 0.30 | 0.70 | 0.001 |

**File name: Additional file 2**

File format: figure

**Figure S1.** The number of community classes as a function of sample size. 50,100,150 and 180 samples were randomly sampled from the total 209 stool samples. Each process was repeated for 100 times. (A). For each subsampling, the optimal number of community classes as determined by the silhouette value in hierarchical clustering was computed. Y-axis indicates the probability of two, three, four, five cluster as the optimal number of clusters among 100 times’ of subsampling. (B). Average silhouette value at 2, 3, 4, 5 clusters at different sample size.

**Figure S2.** PCoA illustrating three stool enterotypes of HMP data using k-means clustering and Jensen-Shannon distance. Using this clustering approach, three enterotypes were identified with SI=0.17.

**Figure S3.** Silhouette plot of different number of clusters of right retroauricular crease. SI value in each subgroup and the average SI values were taken into account to assistant in determining the optimal number of clusters when the average SI is very similar in different partitions.

**Figure S4.** Community classes in posterior fornix at strain level resolution. Six community classes were identified using the WGS data set. Community class 1 and 2 are both dominant by *L. jensenii* species, however, the strain compositions are different in community class 1 and 2.

**Figure S5.** Association of Shannon diversity with average SI. Average SI of the optimal number of cluster has strong negative correlation with alpha diversity measured by Shannon index.

**Figure S6. Community classes and demographic data mapping in hierarchical clustering.** Hierarchical clustering was performed based on Bray-Curtis distance and complete linkage. Gender, geographical location, race, and BMI were mapped along the 18 dendrograms. The top 25 abundant genera of each habitat were plotted along with the cluster. Significantly uneven distribution of meta data were found as below by Chi-square or Fisher’s exact test: Uneven distribution of gender in anterior nares, retroauricular crease, antecubital fossa, stool; PH difference in two community classes of vagina; Age difference in retroauricular crease, keratinized gingiva, hard palate; Uneven distribution of geographical location, ethnicity in two groups of palatine tonsils. Uneven distribution of geographical location, ethnicity in two groups of saliva. Uneven distribution of geographical location in two groups of subgingival plaque. Uneven distribution of geographical location ethnicity in two groups of supragingival plaque. Uneven distribution of geographical location, BMI, ethnicity in two groups of throat.

**Figure S7. Gender differences in the abundance of the representative organisms from community classes.** Boxplots show the relative abundances of each organism in all of the subjects at (A) retroauricular crease (B) antecubital fossa and (C) anterior nares. The asterisks on top of the figure indicate statistically significant differences in the relative abundance between males and females. (D) In anterior nares, *Moraxella* abundances are significantly different for males and females who have at least one *Moraxella* read. (E). In stool, *Prevotella* abundances are significantly different for males and females who have at least one *Prevotella* read.

**Figure S8. Community classes stability over time in vagina, antecubital fossa.** The scheme of the graphs in (A) vagina and (B) anticubital fossa is similar as described in Figure 6. (A) Majority of the subjects maintains in the Lactobacillus community in the second visit. (B). Community classes in antecubital fossa are under active switching especially with the smaller community classes switching to the major community class (*Propinionibacterium*).
