# Supplementary material for: Exploration of bacterial community classes in major human habitats
Source: Genome Biol. 2014 May 7;15(5):R66. doi: 10.1186/gb-2014-15-5-r66 (PMC4073010; doi:10.1186/gb-2014-15-5-r66)
Supplement: Additional file 2 — Figures S1 to S8. [file gb-2014-15-5-r66-S2.pptx]

## Slide 1
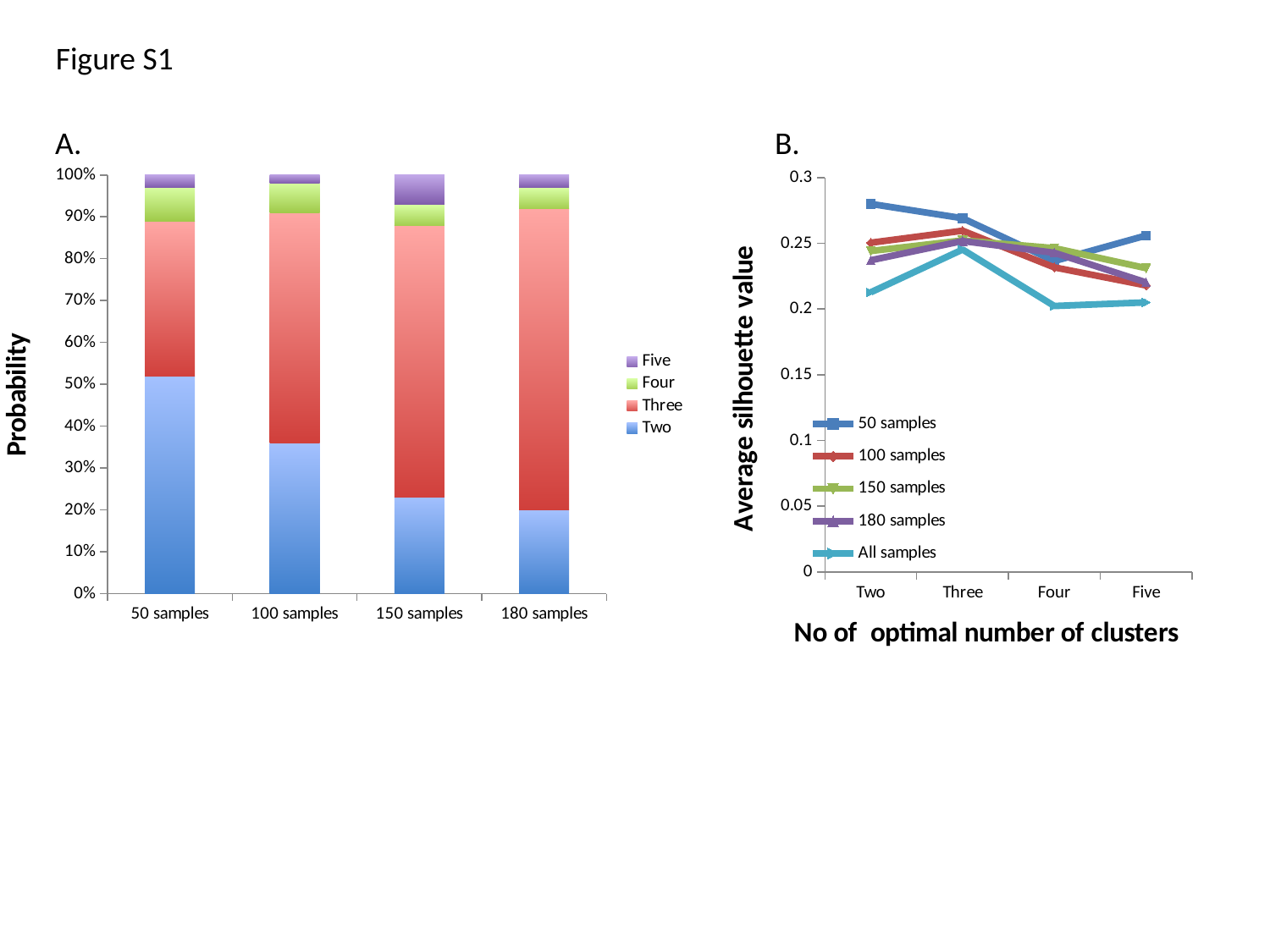

Figure S1
A.
B.
### Chart
| Category | Two | Three | Four | Five |
|---|---|---|---|---|
| 50 samples | 52.0 | 37.0 | 8.0 | 3.0 |
| 100 samples | 36.0 | 55.0 | 7.0 | 2.0 |
| 150 samples | 23.0 | 65.0 | 5.0 | 7.0 |
| 180 samples | 20.0 | 72.0 | 5.0 | 3.0 |
### Chart
| Category | 50 samples | 100 samples | 150 samples | 180 samples | All samples |
|---|---|---|---|---|---|
| Two | 0.2800337 | 0.2504934 | 0.2441641 | 0.2371463 | 0.212782265 |
| Three | 0.2691019 | 0.25961 | 0.2522415 | 0.2517887 | 0.245321801 |
| Four | 0.2365282 | 0.2317416 | 0.2463798 | 0.2428392 | 0.202348799 |
| Five | 0.2559699 | 0.2179816 | 0.2311383 | 0.2203227 | 0.205110436 |

## Slide 2
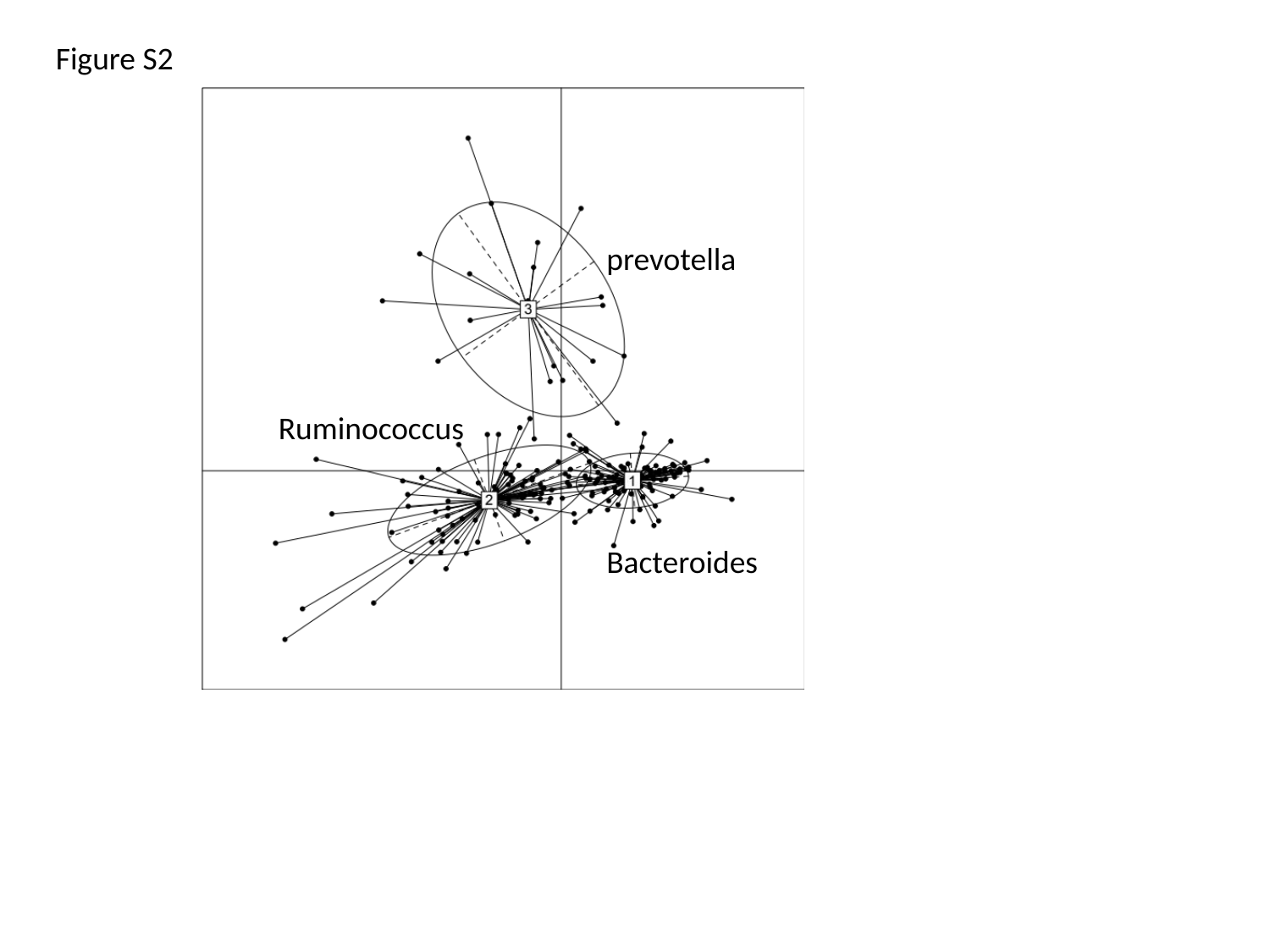

Figure S2
prevotella
Ruminococcus
Bacteroides

## Slide 3
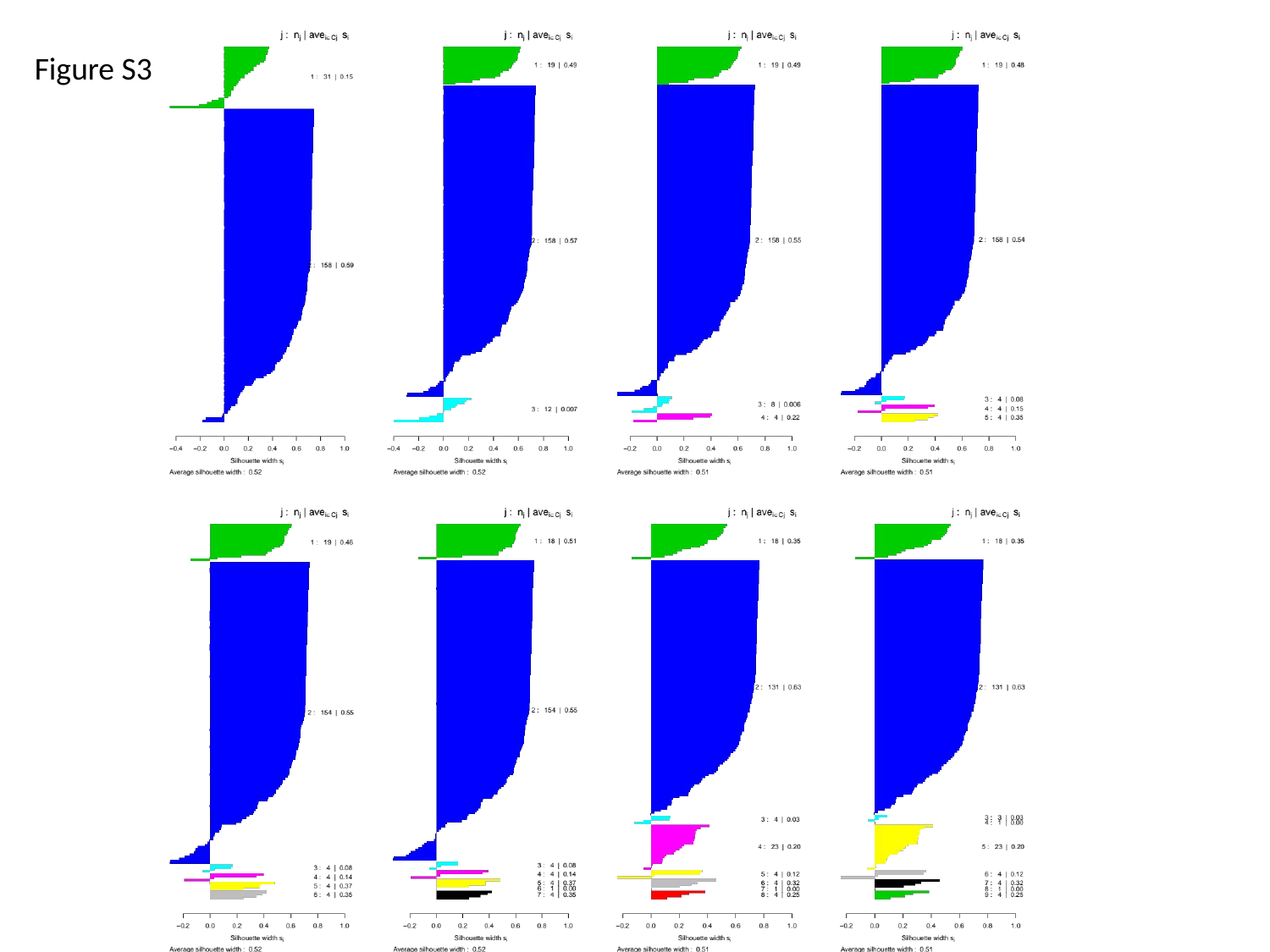

Figure S3

## Slide 4
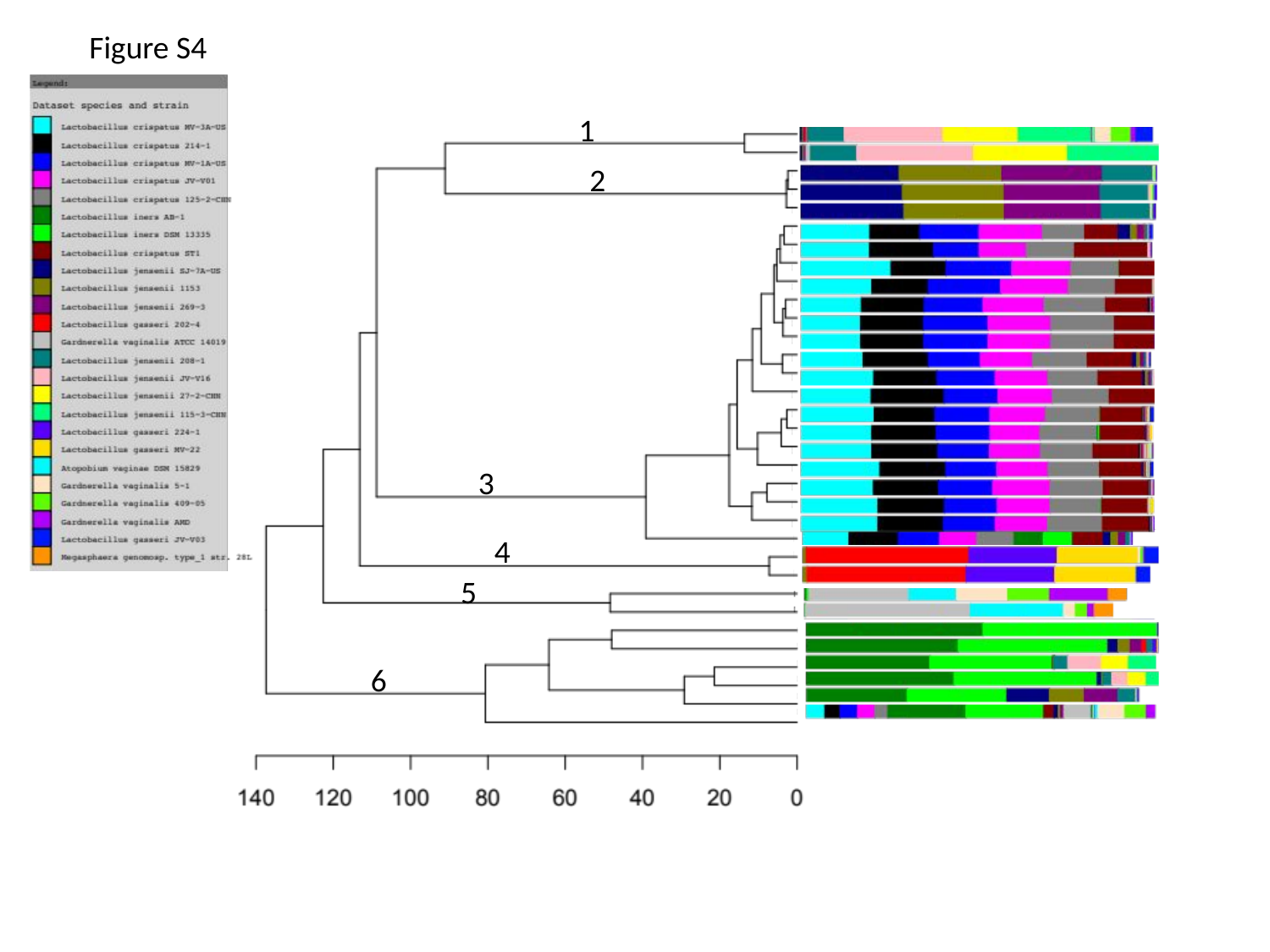

Figure S4
1
2
3
4
5
6

## Slide 5
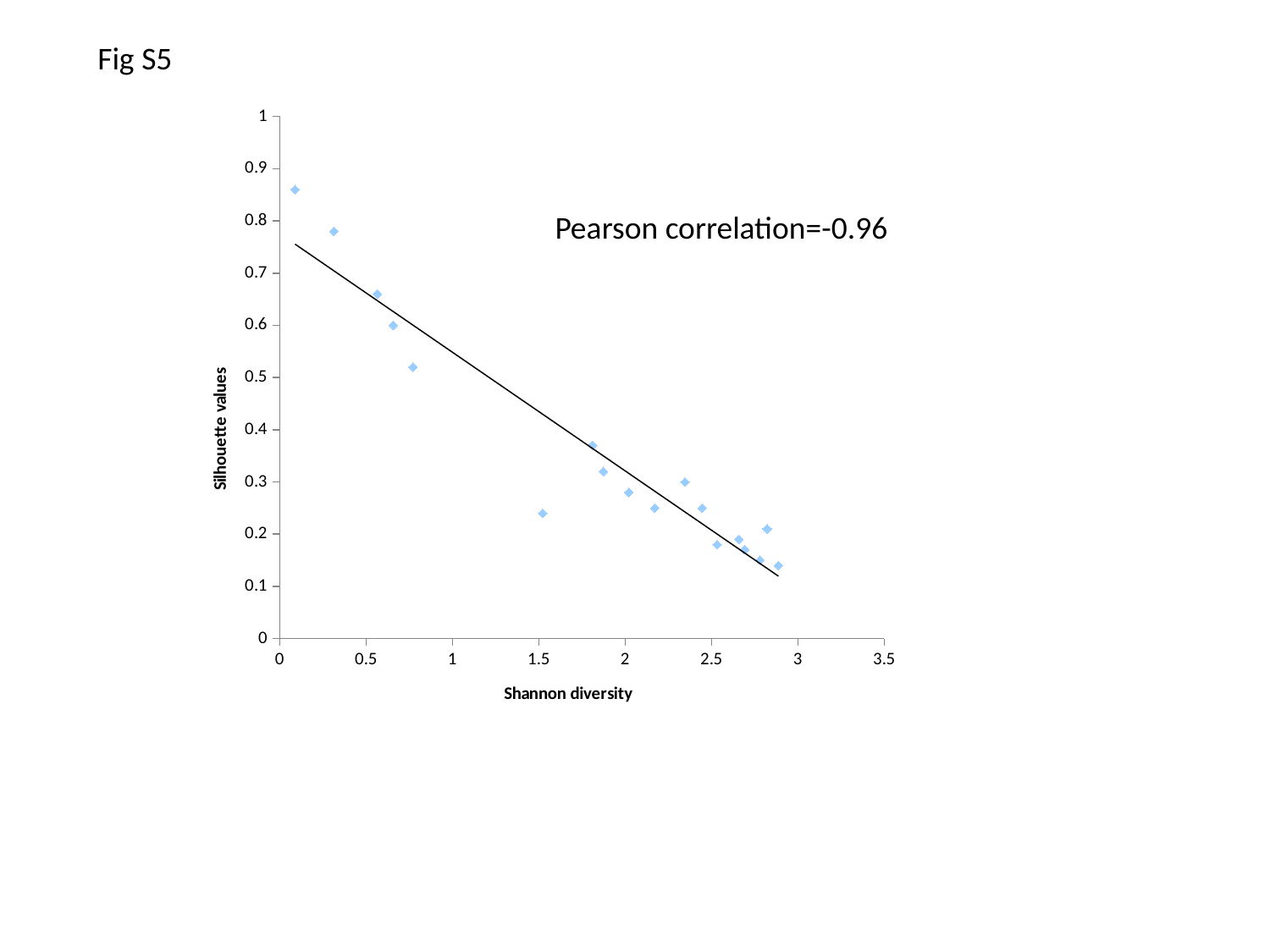

Fig S5
### Chart
| Category | |
|---|---|Pearson correlation=-0.96

## Slide 6
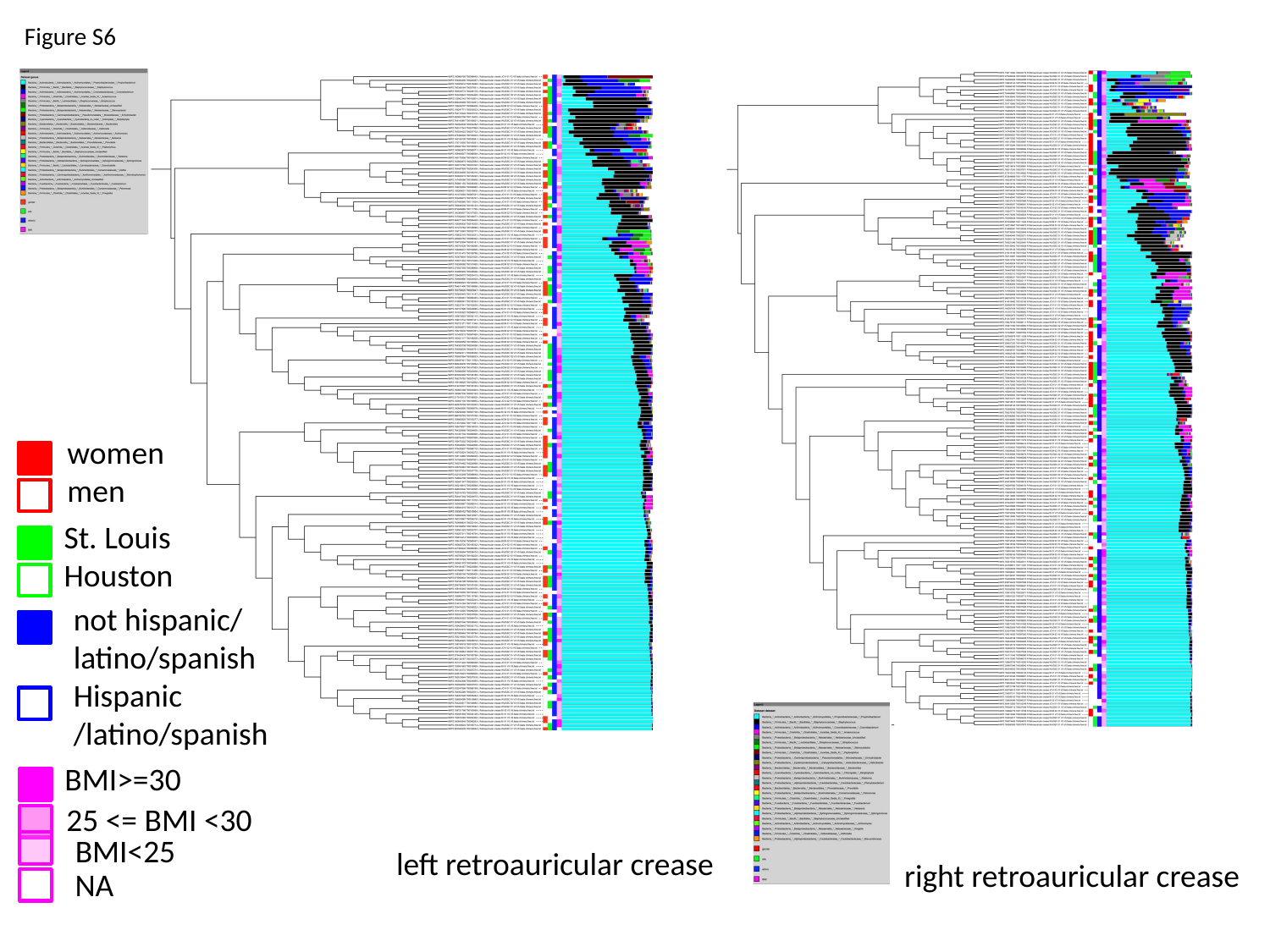

Figure S6
women
men
St. Louis
Houston
not hispanic/
latino/spanish
Hispanic
/latino/spanish
BMI>=30
25 <= BMI <30
BMI<25
left retroauricular crease
right retroauricular crease
NA

## Slide 7
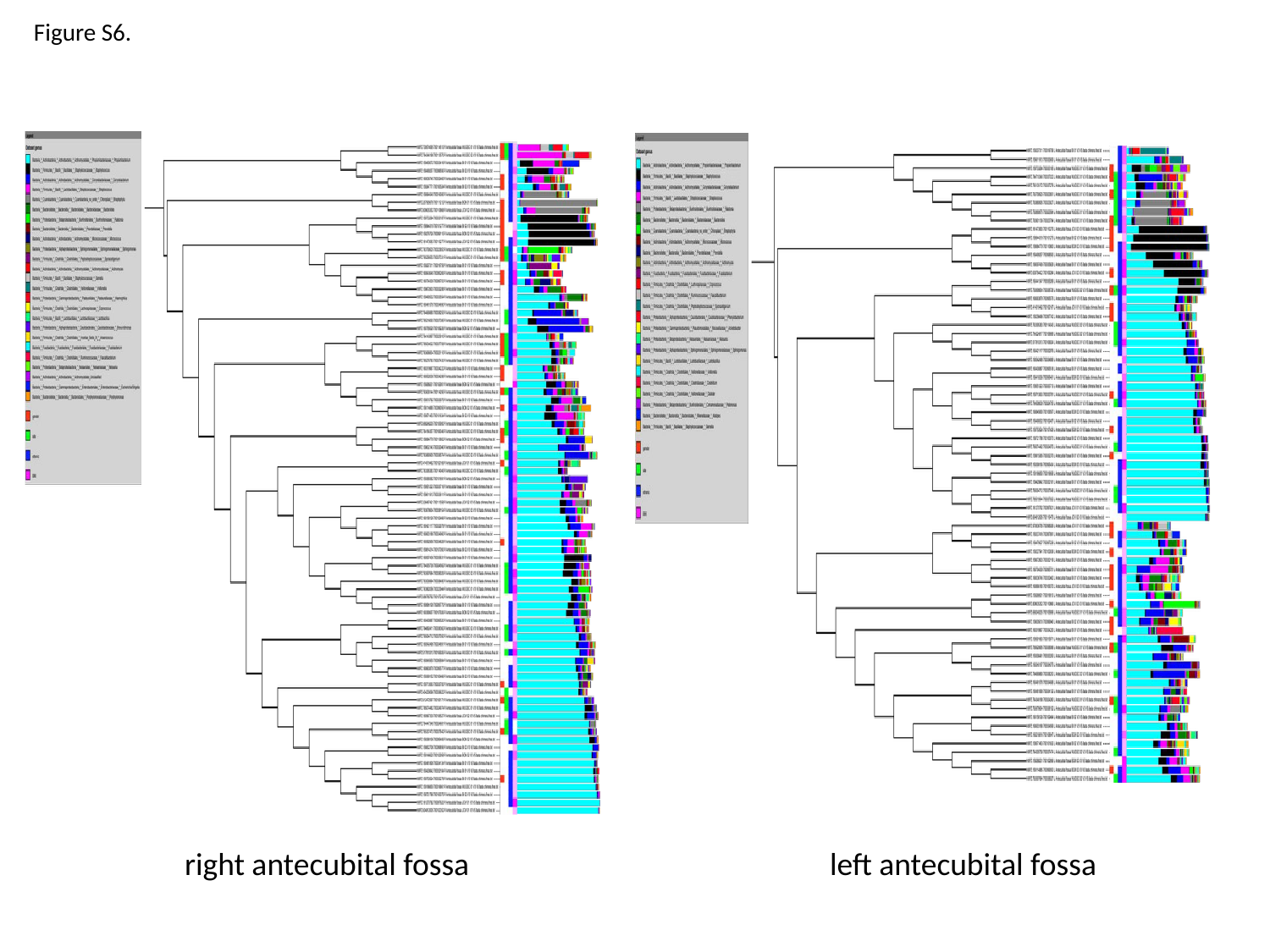

Figure S6.
right antecubital fossa
left antecubital fossa

## Slide 8
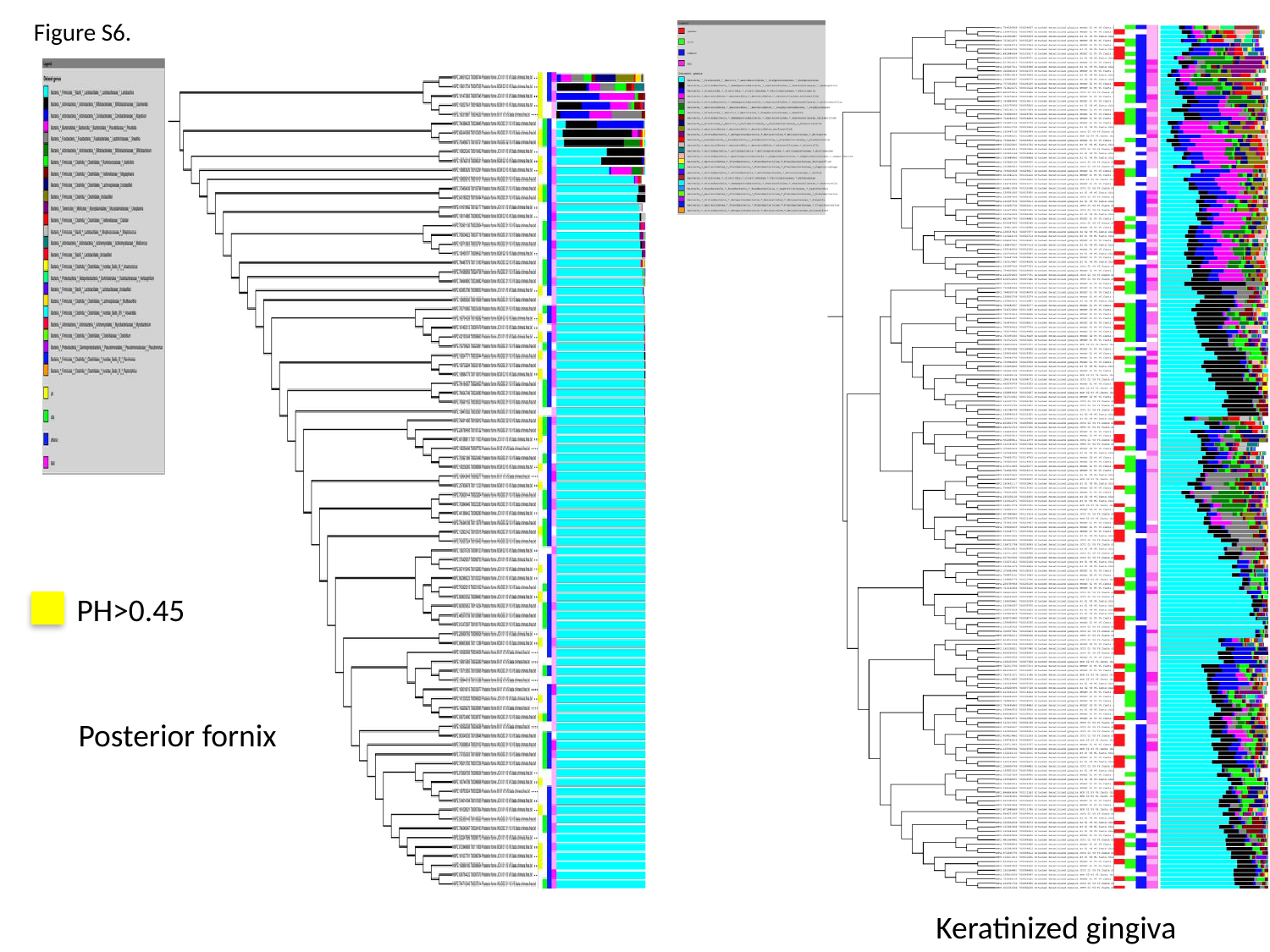

Figure S6.
PH>0.45
Posterior fornix
Keratinized gingiva

## Slide 9
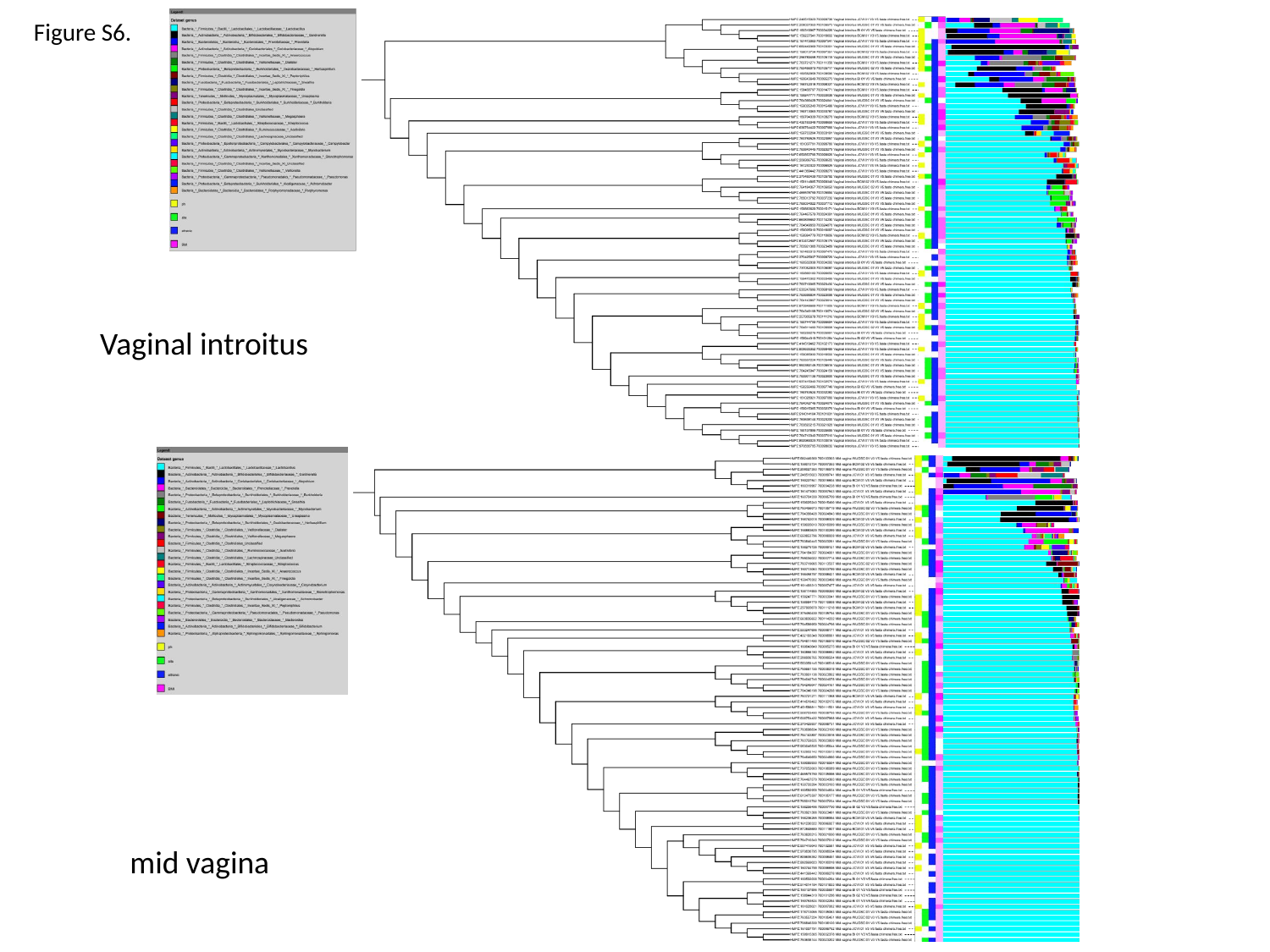

Figure S6.
Vaginal introitus
mid vagina

## Slide 10
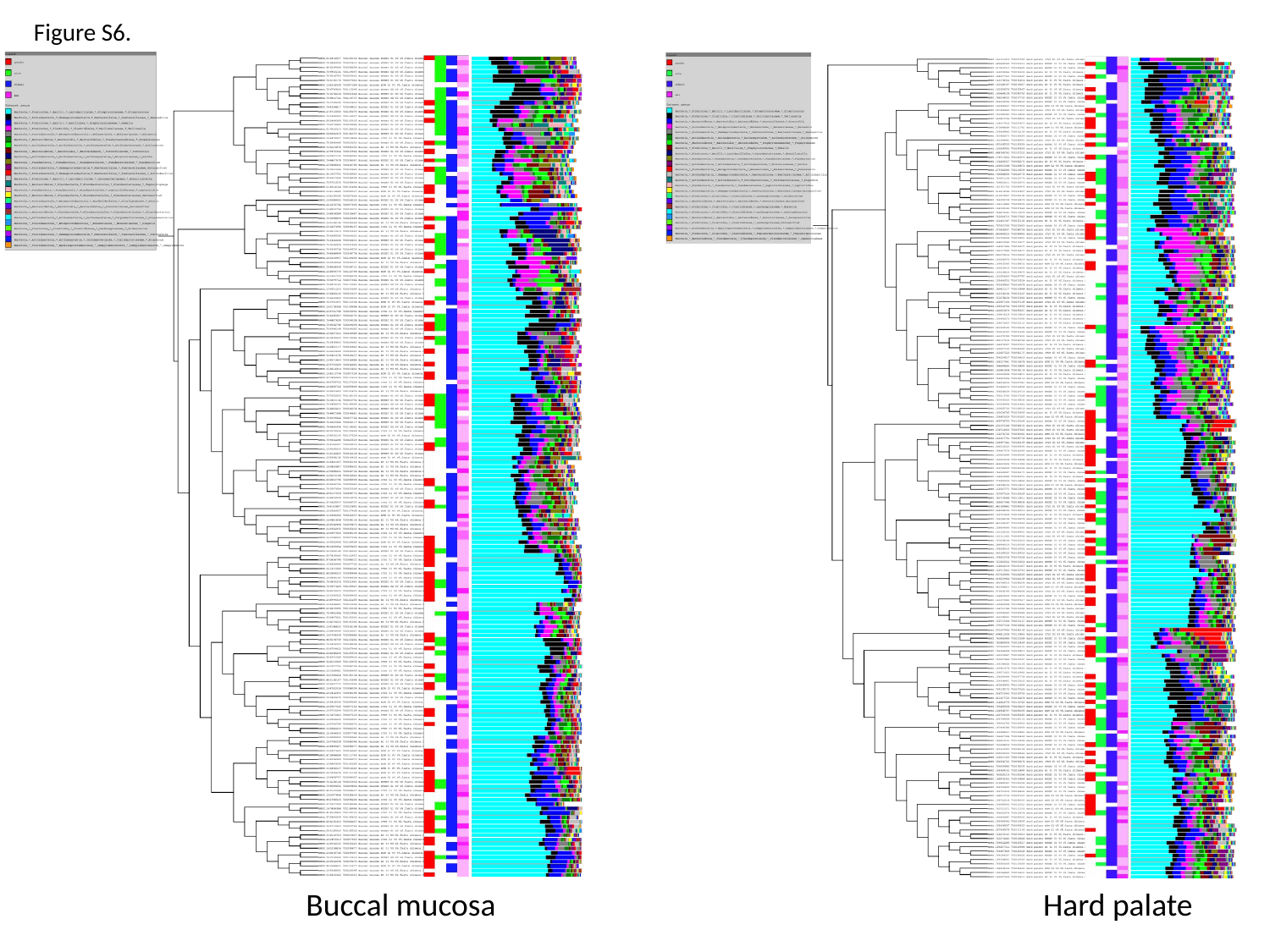

Figure S6.
Buccal mucosa
Hard palate

## Slide 11
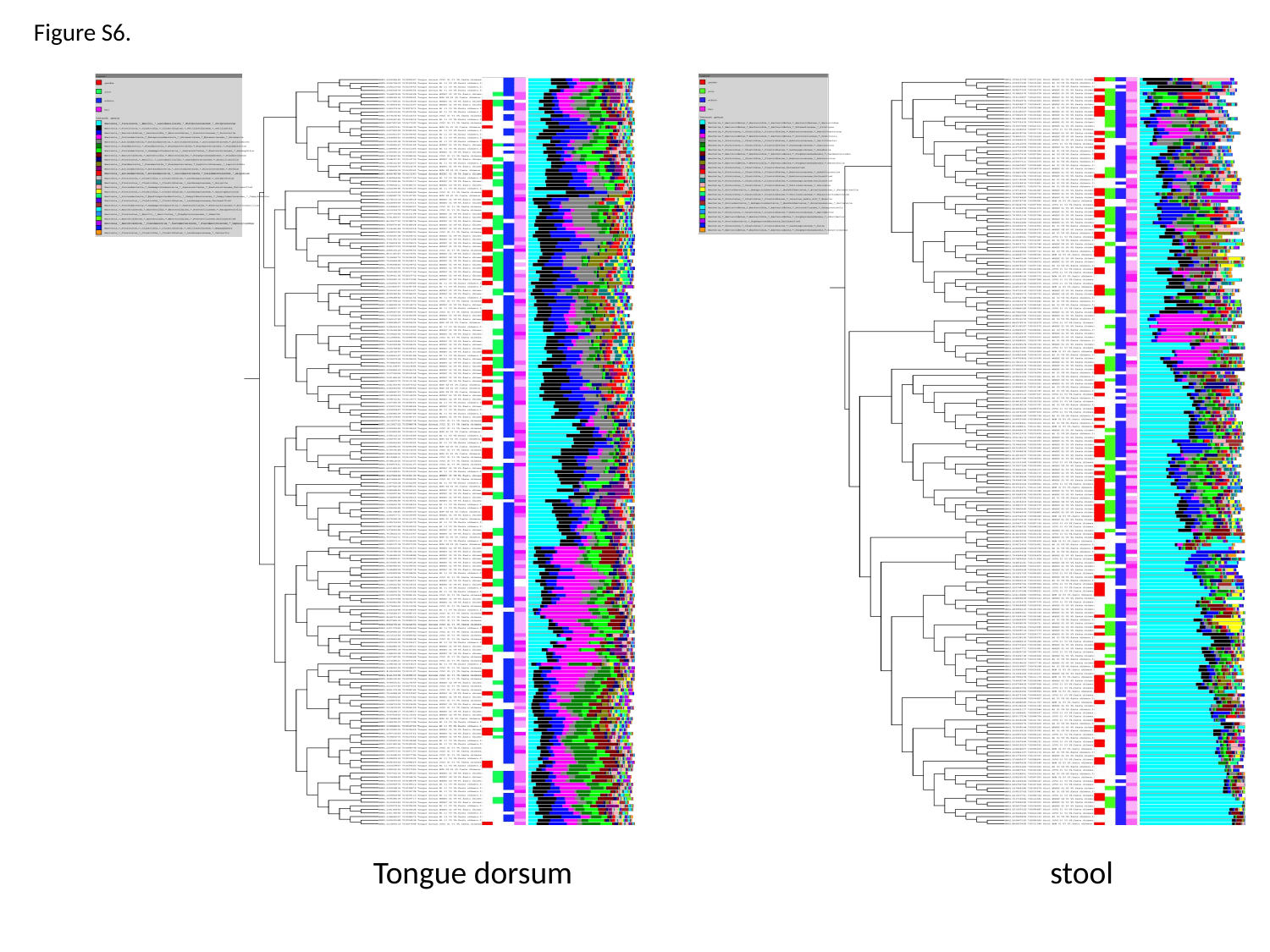

Figure S6.
Tongue dorsum
stool

## Slide 12
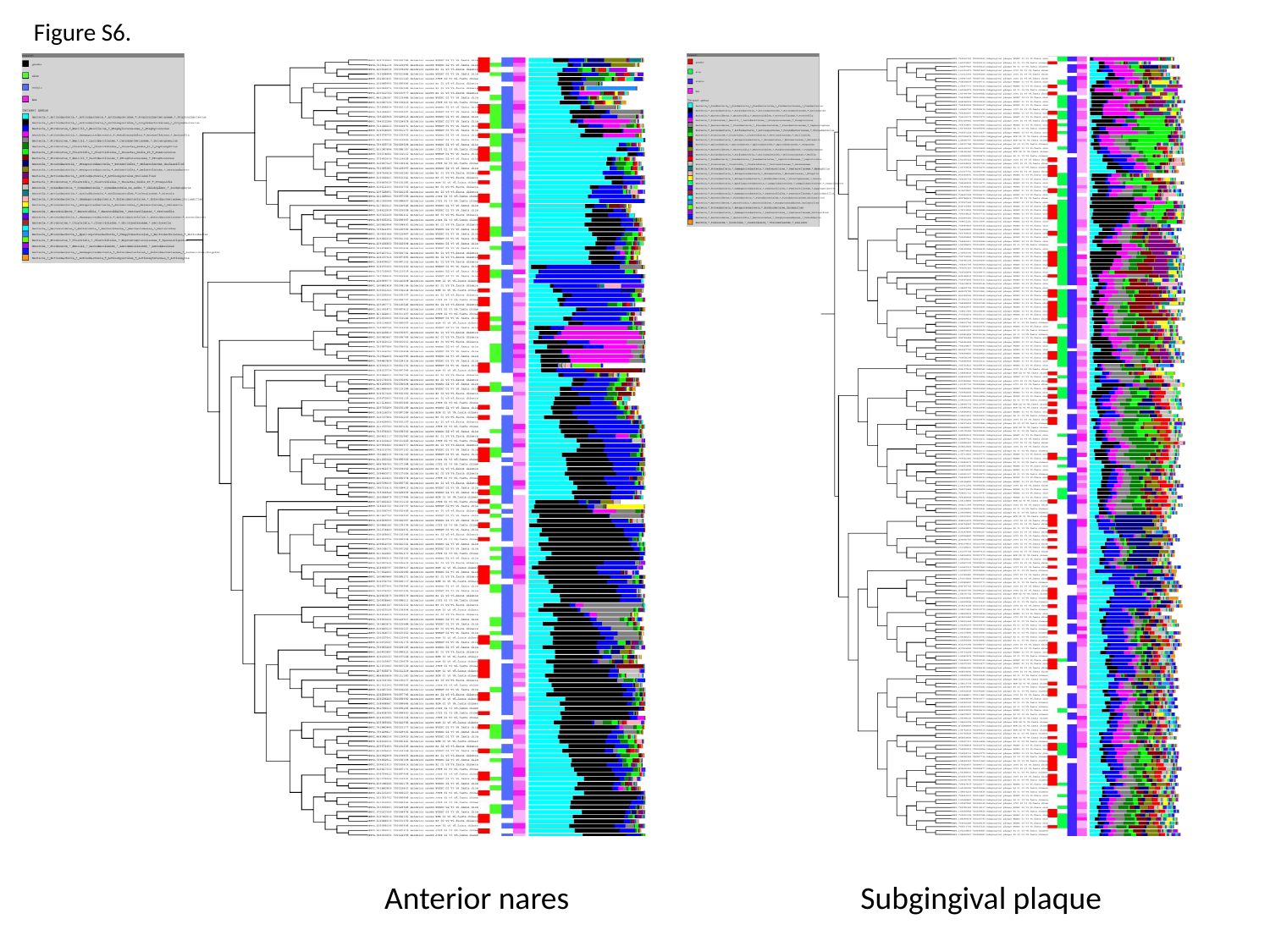

Figure S6.
Anterior nares
Subgingival plaque

## Slide 13
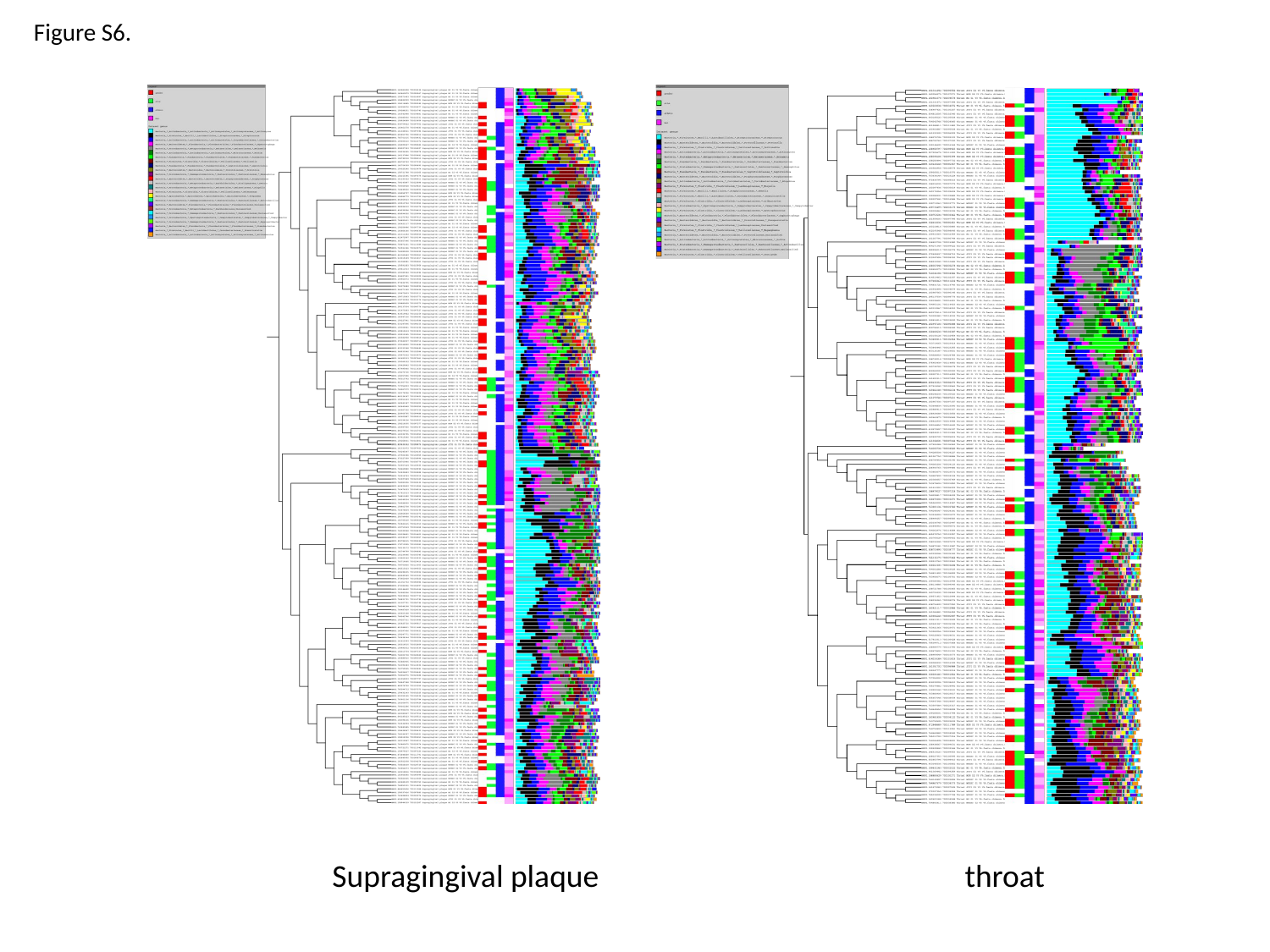

Figure S6.
Supragingival plaque
throat

## Slide 14
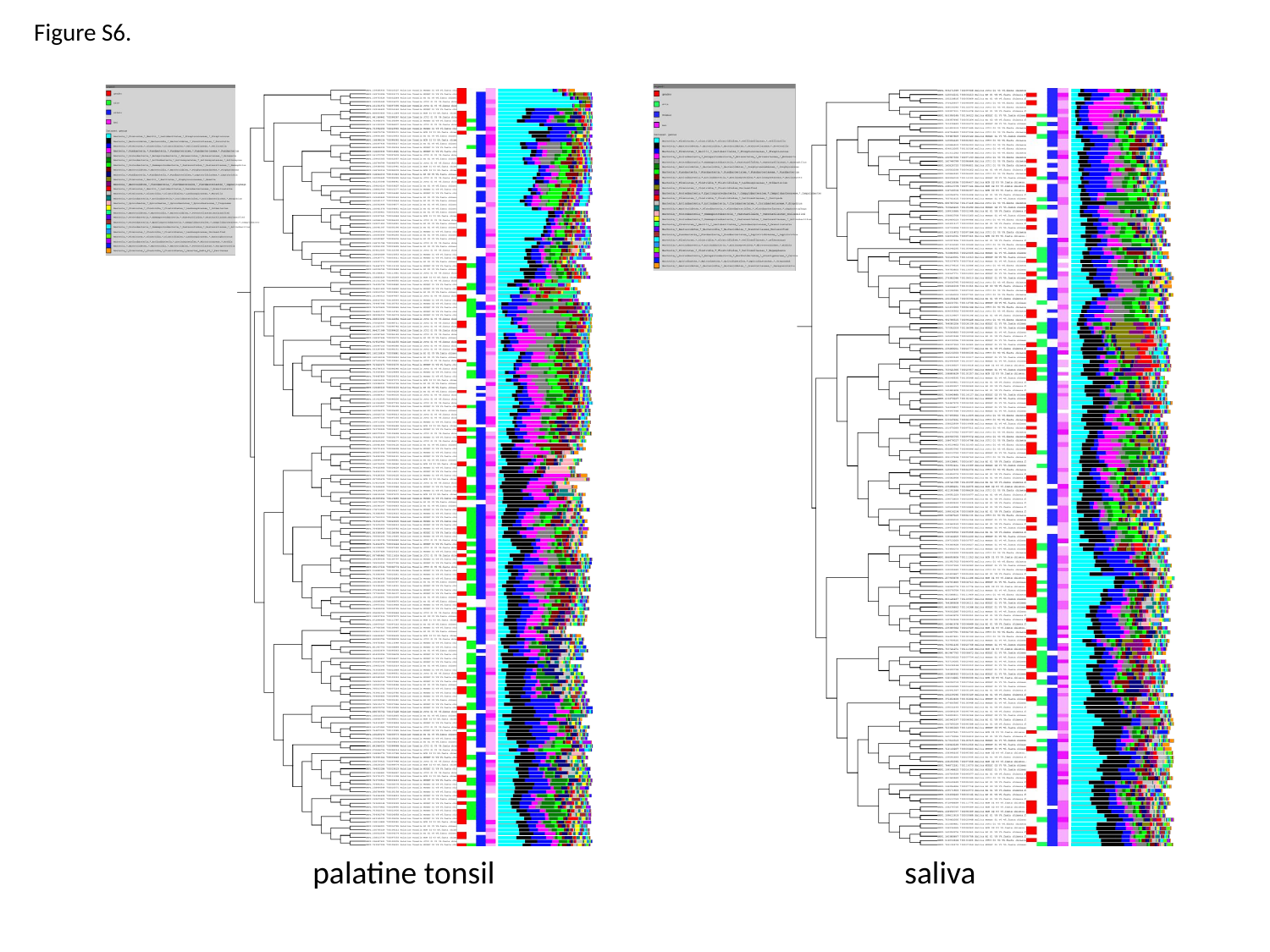

Figure S6.
palatine tonsil
saliva

## Slide 15
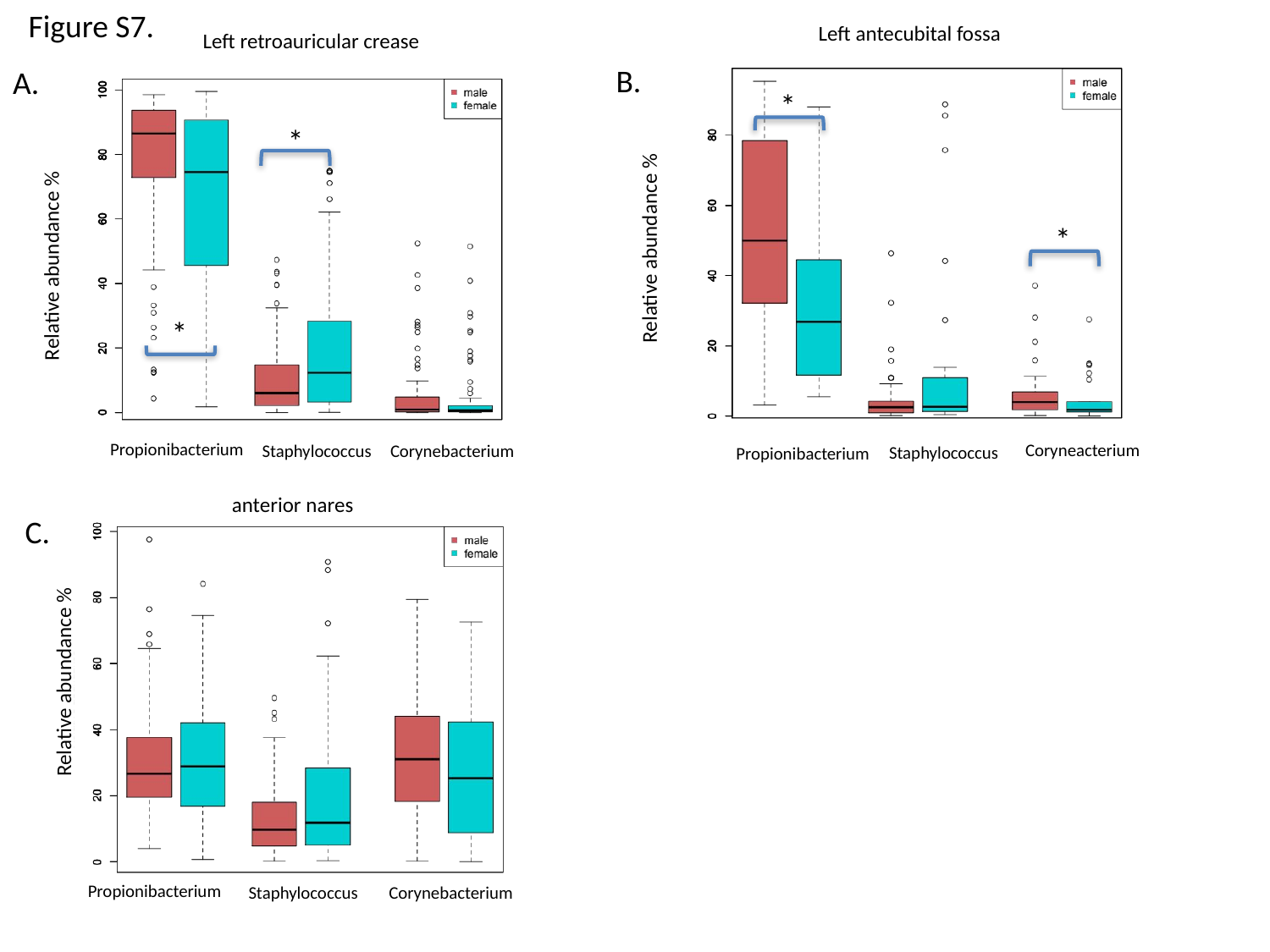

Figure S7.
Left antecubital fossa
Left retroauricular crease
B.
A.
*
*
*
Relative abundance %
Relative abundance %
*
Propionibacterium
Coryneacterium
Staphylococcus
Corynebacterium
Staphylococcus
Propionibacterium
anterior nares
C.
Relative abundance %
Propionibacterium
Staphylococcus
Corynebacterium

## Slide 16
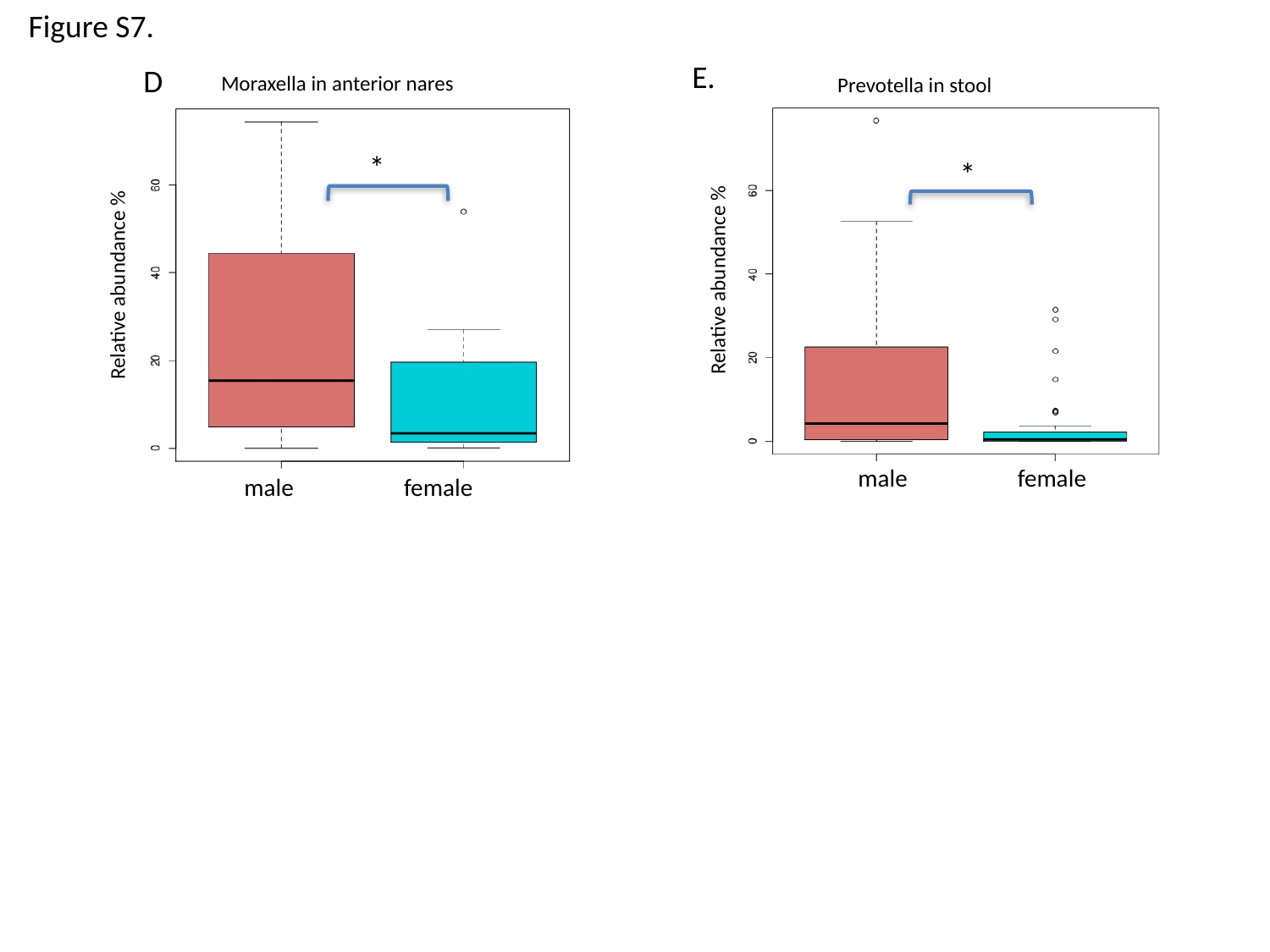

Figure S7.
E.
D
Moraxella in anterior nares
Prevotella in stool
*
*
Relative abundance %
Relative abundance %
male
female
male
female

## Slide 17
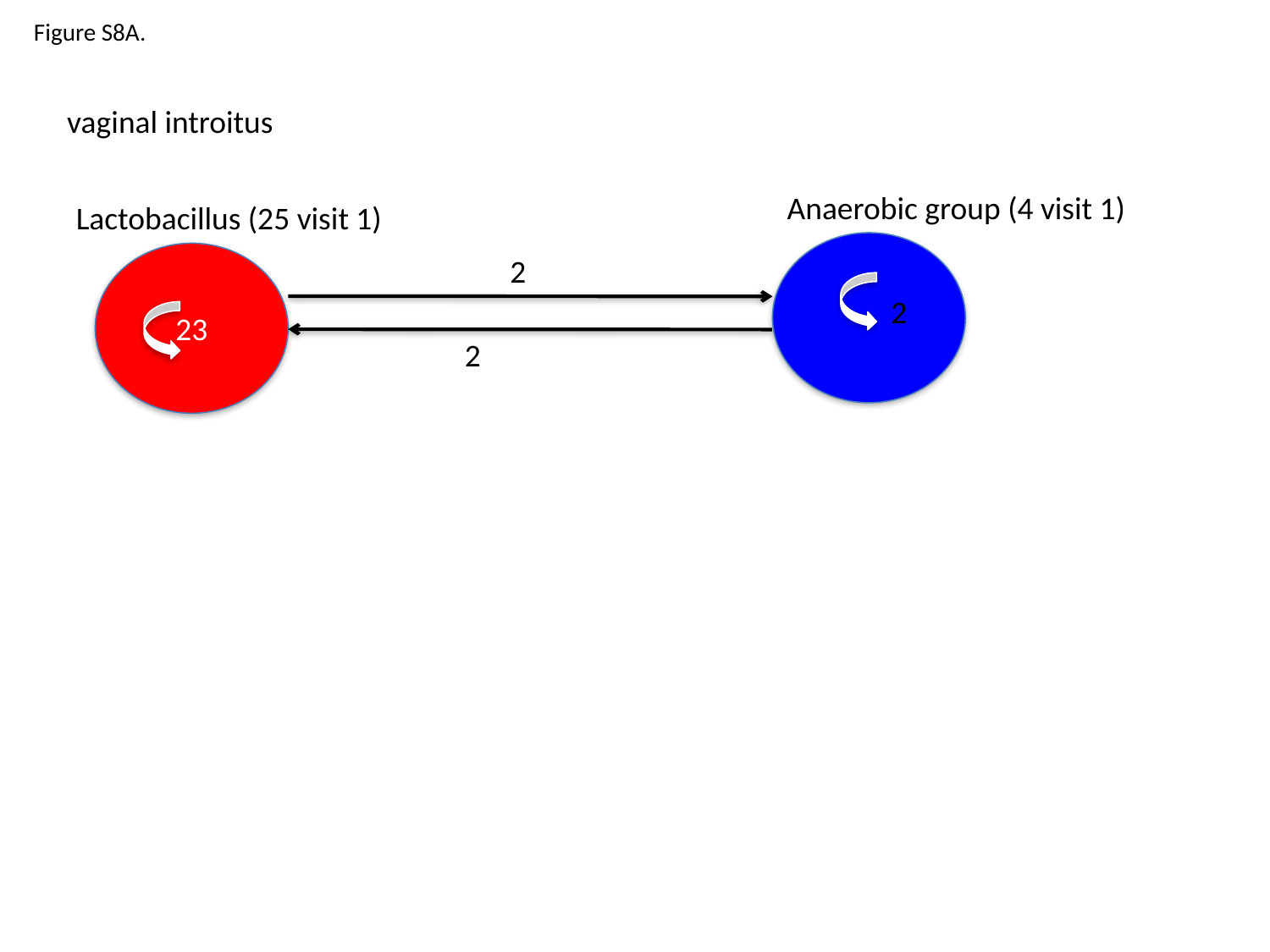

Figure S8A.
vaginal introitus
Anaerobic group (4 visit 1)
Lactobacillus (25 visit 1)
23
2
2
2

## Slide 18
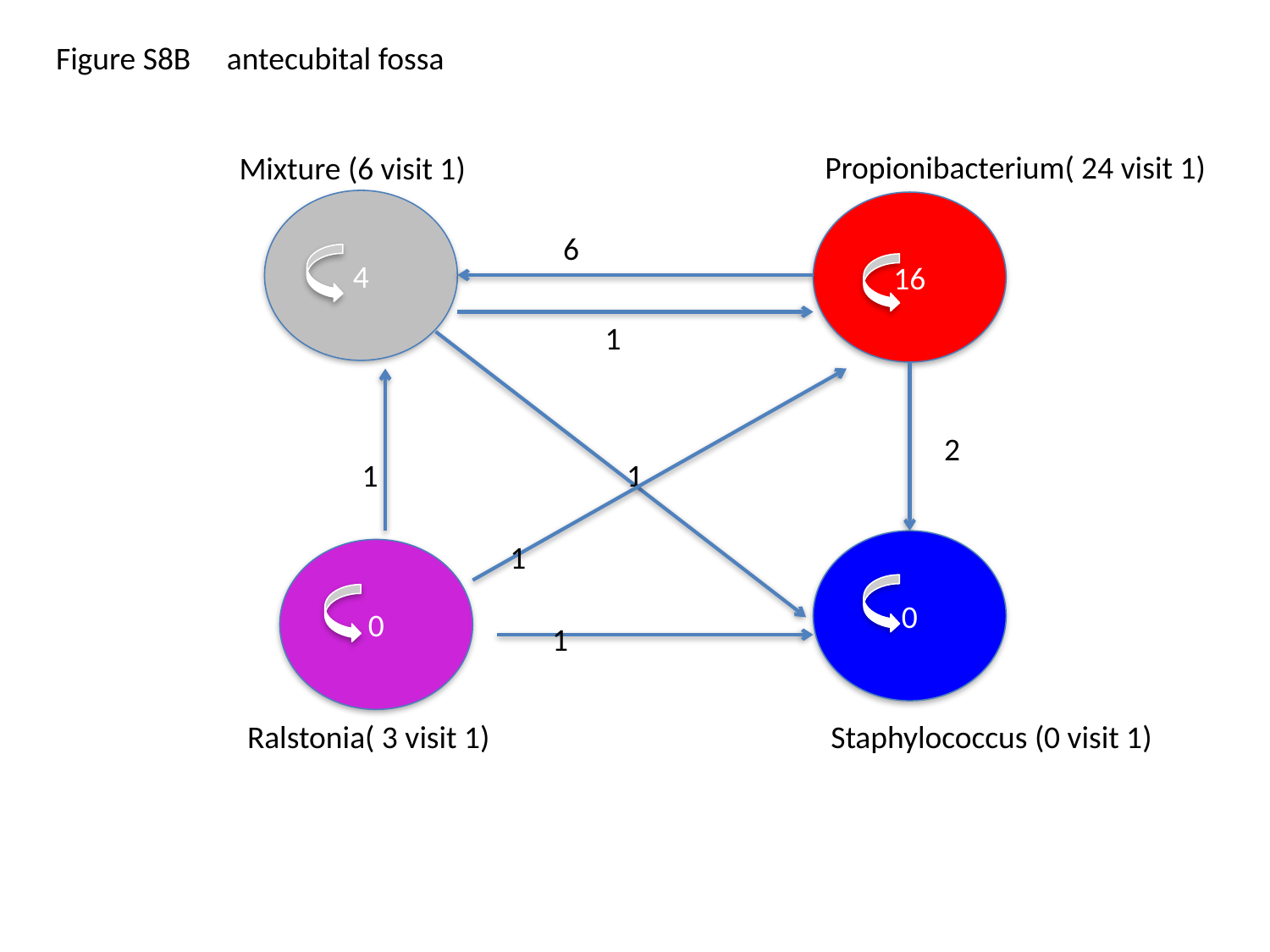

Figure S8B
antecubital fossa
Propionibacterium( 24 visit 1)
Mixture (6 visit 1)
4
16
6
1
2
1
1
1
0
0
1
Ralstonia( 3 visit 1)
Staphylococcus (0 visit 1)
